# Characterization of Tiroler Bergkäse PDO cheese: A multimethodological approach

**DOI:** 10.1016/j.fochms.2025.100336

**Published:** 2025-12-08

**Authors:** Hannah Innerbichler, Alexander Trockenbacher, Alexander Höller, Sabine Scholl-Bürgi, Lorenzo Del Vecchio, Martina Cirlini, Jürgen König, Katrin Bach

**Affiliations:** a Department of Food Technology & Nutrition, MCI | The Entrepreneurial School, 6020 Innsbruck, Austria; b Department of Nutritional Sciences, University of Vienna, 1010 Wien, Austria; c Department of Biotechnology & Food Engineering, MCI | The Entrepreneurial School, 6020 Innsbruck, Austria; d Division of Nutrition and Dietetics, University Hospital Innsbruck, 6020 Innsbruck, Austria; e Department of Child and Adolescent Health, Division Pediatrics I, Medical University of Innsbruck, 6020 Innsbruck, Austria; f Department of Food and Drug, University of Parma, 43124 Parma, Italy

**Keywords:** Tiroler Bergkäse, Hard cheese, Protected designation of origin (PDO), Multimethodological characterization, Quantitative polymerase chain reaction (qPCR), Ion exchange chromatography (IEC), Headspace-solid phase microextraction/gas chromatography-mass spectrometry (HS-SPME/GC-MS), Multivariate analysis

## Abstract

Tiroler Bergkäse PDO is a traditional Austrian hard cheese from Tyrol, a product of great regional significance. This study aimed to describe Tiroler Bergkäse PDO by comparing it with Bergkäse without PDO from Tyrol and a Stilfser type cheese from South Tyrol. To account for variability, three cheese wheels from three production days were analyzed for each cheese. Multimethodological characterization included quantitative Polymerase Chain Reaction, Ion Exchange Chromatography, and Headspace-Solid Phase Microextraction/Gas Chromatography-Mass Spectrometry. The characterization Tiroler Bergkäse PDO should impart potentially unique features. Multivariate analysis of the bacterial composition, free amino acid content, and volatile fraction revealed differences between all cheeses (α = 0.05). A triangle test confirmed significant differences, with more pronounced differences between the Bergkäse type cheeses and the Stilfser type cheese. The Stilfser type showed distinct differences in bacterial composition, amino acid content, and volatile profile, making it less similar to the Bergkäse type cheeses. Multivariate analysis also revealed differences between the PDO and non-PDO Bergkäse which is characterized by high levels of L. *delbrueckii* due to its starter culture, and a high free amino acid content from longer ripening, alongside elevated levels of hexanal and acetoin. In contrast, Bergkäse without PDO shows high levels of 2,3-butanediol and 2-nonanone, alongside high levels of L. *mesenteroides*, *L. lactis* subsp. *lactis*, and L. *lactis* subsp. *cremoris*. It was possible to distinguish between Tiroler Bergkäse PDO from non-PDO cheeses by microbial, amino acid, and volatile profiles and support the use of advanced methods to characterize regional foods.

## Highlights

• Multimethodology to characterize alpine cheeses and distinguish between PDO and non-PDO.
• qPCR, IEC, HS-SPME/GC–MS, and multivariate analysis used.
• Microbial, amino acid, and volatile profiles confirmed differences between cheeses.
• Multimethodological analysis linked microbial diversity to PDO cheese quality.
• PDO Bergkäse's traits were shaped by traditional production methods.

## 1 Introduction

Tiroler Bergkäse PDO is a traditional Austrian hard cheese whose cultural, historical, and economic relevance is well documented; however, unlike many other Protected Designation of Origin (PDO) cheeses – such as Parmigiano Reggiano, Grana Padano, Mozzarella di Bufala Campana, or Serra da Estrela – its molecular, biochemical, and microbial characteristics have not been comprehensively described using multimethodological approaches. While several PDO cheeses have been extensively profiled using advanced analytical tools, similar studies for Tiroler Bergkäse PDO are scarce. Therefore, a clear knowledge gap exists regarding the integrated characterization of its microbial, proteolytic, and volatile profiles and how these compare not only to similar regional cheeses but also to non-PDO alternatives. This study addresses this gap by applying qPCR, ion exchange chromatography (IEC), and HS-SPME/GC–MS to provide the first multimethodological characterization of Tiroler Bergkäse PDO.

The production of Tiroler Bergkäse dates back to the 1840s, when long-lasting cheese varieties were needed to transport Tyrolean milk products to distant markets such as Vienna and Berlin (Bundesministerium Land- und Forstwirtschaft, Regionen und Wasserwirtschaft, 2024; Tiroler Milchkäuferverband, 1997). The traditional production practices, including high curd-heating temperatures and extended ripening, have been preserved and were formally recognized in 1997 when Tiroler Bergkäse received PDO certification (Tiroler Milchkäuferverband, 1997; European Commission, 1997). This certification protects traditional knowledge, supports rural economies, and ensures adherence to region-specific manufacturing methods, thus playing a significant role in the regional economy (Savelli, Bravi, Francioni, Murmura, & Pencarelli, 2021).

PDO regulations stipulate that Tiroler Bergkäse must be produced in Tyrol using raw hay milk, calf rennet, high-temperature curd heating (approx. 52 °C), brining in 20 % NaCl solution, and ripening under controlled humidity and temperature for at least three months. Notably, the specification does not mandate a specific starter culture composition, which remains at the discretion of the producer. This contributes to natural variability among cheeses and underscores the importance of analytical characterization.

PDO cheeses can often be differentiated based on their microbiota, proteolytic activity, and volatile profiles. While extensive studies exist for well-established PDO cheeses, similar analyses for Tiroler Bergkäse PDO are lacking. Understanding these parameters not only strengthens product authentication strategies but also contributes to the preservation of traditional production practices.

The influence of PDO labeling on consumer perception and sensory experience has been previously investigated, demonstrating a significant halo effect on Tiroler Bergkäse PDO (Innerbichler, Danzl, König, & Bach, 2024). While that study focused on the sensory experience and perception, the present research aims to complement these findings by providing a characterization of Tiroler Bergkäse PDO compared to non-PDO Bergkäse and a Stilfser type cheese. It is imperative to characterize traditional products, ensuring continued support for these regional economies.

Three complementary analytical approaches were chosen in the present study: (i) qPCR enables targeted quantification of key microbial taxa and is particularly suited for differentiating cheeses with distinct starter cultures or raw milk microbiota; (ii) IEC provides detailed profiles of free amino acids, which reflect proteolysis, starter activity, and ripening time – parameters highly relevant for distinguishing PDO vs. non-PDO cheeses; and (iii) HS-SPME/GC–MS identifies and semi-quantifies volatile compounds responsible for sensory attributes and can reflect differences in microbial metabolism and ripening.

These methods have been successfully used in molecular profiling of other PDO cheeses. Villanueva et al. for example used qPCR to identify adulteration of goat cheese in Mexico (Villanueva-Zayas, Rodríguez-Ramírez, Villa-Lerma, et al., 2024) and were able to identify a high degree of bovine milk use for the production of goat cheese. Pellegrino et al. combined proteomic parameters including IEC to authenticate grated Grana Padano PDO cheese (Pellegrino, Rosi, Sindaco, and D'Incecco, 2024). Italian Pecorino cheeses were successfully discriminated using HS-SPME/GC–MS for the analysis of volatiles, despite their production occurring in different production cycles and in nearby geographical areas (Di Donato, Biancolillo, Mazzulli, Rossi, & D'Archivio, 2021). The combined application of these methods may allow a multidimensional differentiation between Tiroler Bergkäse PDO, non-PDO Bergkäse, and Stilfser-type cheese.

This study is one of the first aimed to characterize Tiroler Bergkäse PDO, a niche product of great regional significance to their unique features. This study used three methods – qPCR for microbial characterization, IEC with post-column ninhydrin derivatization to determine the FAA contents, and headspace solid-phase microextraction coupled with gas chromatography–mass spectrometry (HS-SPME/GC–MS) to examine the volatiles – combined with multivariate analysis to characterize Tiroler Bergkäse PDO by comparing it to the similar Bergkäse without PDO, also produced in Tyrol, as well as from a Stilfser type cheese produced in South Tyrol, Italy. The novelty of the present research lies in its ability to not only characterize Tiroler Bergkäse PDO but also to establish a framework for understanding how traditional production methods and regional specificity contribute to the uniqueness of PDO cheeses.

## 2 Material and methods

### 2.1 Sample set

In total, 27 commercial cheese samples were purchased from two manufacturers in Tyrol, Austria, and one in South Tyrol, Italy. Table 1 lists the basic information on the cheese samples included in this study.

**Table 1:** Sample information.

Cheese type	Sample Abbreviation	Date of manufacture	Ripening time (days)	Milk type
Bergkäse w/o PDO	A-C BK	15 Oct 22	107	Tyrolean raw-hay milk^a^
	D-F BK	22 Nov 22	107	
	G-I BK	16 Dec 22	112	
Tiroler Bergkäse PDO	A-C BKGU	01 Oct 22	119	Tyrolean raw-hay milk^a^
	D-F BKGU	31 Oct 22	123	
	G-I BKGU	02 Dec 22	125	
Stilfser type w/o PDO	A-C BATO	24 Nov 22	59	South Tyrolean pasteurized-hay milk^a^
	D-F BATO	20 Dec 22	68	
	G-I BATO	09 Feb 23	64	

Note: ^a^ hay milk is milk produced from cows that are fed primarily with fresh grass, hay, and herbs, without fermented feed like silage (Scheurich et al., 2021).

The sample set included two hard raw milk cheeses: Tiroler Bergkäse PDO and Bergkäse without PDO produced in Tyrol, Austria. Additionally, the sample set contained Stilfser type cheese without PDO, a semi-hard cheese produced in South Tyrol, Italy. Stilfser type without PDO was added to the sample set as an internal control. An internal control was included to ensure that the observed results were due to the true differences in the samples rather than methodological issues. The inclusion of Stilfser type without PDO as an internal control was justified because, due to its distinct characteristics (e.g. milk type, ripening time), it served as a clear reference point for assessing the effectiveness of the techniques in differentiating between cheeses.

A study design of three cheeses, three production days, and three loaves each (3x3x3) was selected valuing biological replicates, which were independently analyzed. The selection of three different production days was made to account for potential day-to-day variations in cheese making, such as fluctuations in raw milk composition and processing conditions. It should be noted, however, that not all aspects are covered since the samples span only a production period from October to February 2022, thereby limiting the generalizability of the findings. Nonetheless, this approach is well-suited for this initial study, as it captures a certain range of variation. Furthermore, the choice of three samples per day was aimed at improving the statistical reliability of the results while remaining feasible in terms of sample processing and analysis.

Tiroler Bergkäse PDO was produced in Tyrol according to its production specification as detailed in the introduction. The Bergkäse without PDO was also produced in Tyrol using Tyrolean hay milk. This cheese differs from Tiroler Bergkäse PDO in three main aspects: the starter culture used, the maturation period, and the type of rennet, which is microbial for the non-PDO Bergkäse cheese. The Stilfser type cheese without PDO was produced with pasteurized hay milk and microbial rennet (Table 1) in South Tyrol. In general, the starter cultures contain *Lactococcus lactis*, *Leuconostoc mesenteroides*, *Streptococcus thermophiles* and *Lactobacillus delbrueckii*. The composition of each cheese specific starter culture will be provided on request. To minimize the impact of starter culture variability, commercially available samples from producers were carefully selected.

**Fig. 1:**
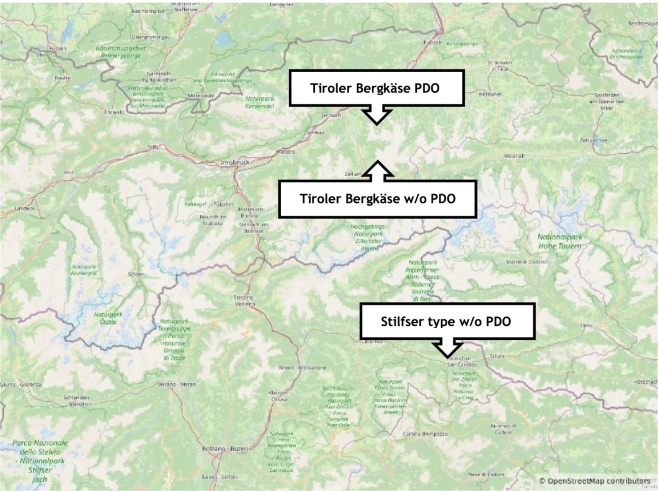
Map of the origin of the cheeses based on https://www.openstreetmap.org.

The linear distance between the manufacturers of Tiroler Bergkäse PDO and Bergkäse without PDO is just 8 km. Consequently, due to the proximity of the production sites of both Bergkäse type cheeses, the use of Isotope Ratio Mass Spectrometry (IRMS) was not feasible. In contrast, the linear distance between the manufacturers of Stilfser type cheese without PDO and Tiroler Bergkäse PDO is 80 km, while the distance between the manufacturers of Stilfser type cheese without PDO and Bergkäse without PDO is 74 km (Fig. 1). All samples belonging to a given cheese type originated from the same manufacturer.

In Austria, alpine and pasture grazing commonly takes place from May to September (Landwirtschaftskammer Niederösterreich, 2025), and is part of traditional dairy farming practices. However, since the cheeses analyzed in this study were produced in October 2022 or later, it is likely that the cows were primarily fed hay at that time. The exact composition of the feeding regime was not explicitly recorded and should be considered as a limitation in interpreting the results.

Cheese samples were obtained from half-loaves and systematically subdivided into twelve segments using a standardized cutting scheme. For analysis, four outer segments were selected, and a 2 cm layer was removed from the rind before storing the samples (see Fig. S1, Supplementary Material for details).

All cheese samples were stored at −80 °C until analysis. For all analyses, the samples were carefully blend and ground into a fine powder. Therefore, three samples from each cheese sample were cut, grated, and weighed (3 g) into a grinding jar containing two grinding balls. The jar was tightly screwed and placed into a liquid nitrogen bath for 4.5 min. The sample was ground for 1.5 min at an oscillating frequency of 30 Hz by the MM 400 mixer mill (Retsch, Haan, Germany). All blended samples from all biological replicates for each cheese type across three production days were analyzed once.

### 2.2 Bacterial composition investigation by QPCR

DNA was extracted from powdered cheese using the NucleoSpin Food DNA purification Kit (Macherey-Nagel, Düren, Germany) according to the supplier's instructions. Genomic DNA was eluted in a volume of 50 μL. The concentration was measured using NanoDrop™ 1000 Spectrophotometer (Thermo Fisher Scientific, Wilmington, USA).

To select the target species for the qPCR analysis the information about the starter cultures for each cheese, provided by the respective manufacturers, was combined with the results of a preliminary experiment, which was conducted to sample and quantify bacterial composition by PCR-amplifying bacterial 16S rDNA with the primer pair: S-D-Bact-0341-b-S-17, S-D-Bact-0785-a-A-21, as suggested by Klindworth et al. (2013) as general 16S rRNA gene PCR primer pair. This amplification produced a mixture of amplicons representing the existing bacterial composition. The amplicons were then separated by cloning using NEB PCR Cloning Kit (New England Biolabs, Ipswich, USA) according to the supplier's instructions, followed by Sanger sequencing of representative clones. Nucleotide sequences were clipped using the Benchling software (Cloud-based platform for biotech R&D, 2024). Clipped sequences were submitted to BLASTn sequence similarity searching and the genera were determined for 47 clones.

The qPCR was performed with 2 ng of the extracted DNA from cheese samples in technical triplicates. All qPCR assays were run on a CFX96 C1000 Touch Thermal Cycler (Bio Rad, Hercules, USA) and analyzed by CFX Maestro Software (Version 2.3–2021, Bio Rad, Hercules, USA). The primer pairs used in this study were purchased from Eurofins Genomics (Eurofins Genomics GmbH, Ebersberg, Germany). The template DNAs of the strains used to create the calibration curves (Supplementary Material, Fig. S2 – S8) were purchased from the Leibniz Institute DSMZ-German Collection of Microorganisms and Cell Cultures (DSMZ, Braunschweig, Germany) (Table 2).

**Table 2:** qPCR overview: Selected targets, primer names, amplicon sizes (bp), and calibration curve strains.

Target	Primer name	Amplicon size (bp)	Strain (Origin of the strain)	Reference
*Lacticaseibacillus casei* group^a^	Lc3 Lc4	121	DSM 20011 (cheese)	(Furet, Quénée, & Tailliez, 2004)
*Lactobacillus delbrueckii*	Lbdelbr_g3904-F Lbdelbr_g3904-R	87	DSM 20072 (emmental cheese)	(Dreier, Berthoud, Shani, Wechsler, & Junier, 2021)
*Lactococcus lactis* subsp. *cremoris*	Lclaccre_mtlD-2F Lclaccre_mtlD-2R	145	DSM 20069 (unknown)	(Dreier et al., 2021)
*Lactococcus lactis* subsp. *lactis*	Lclaclac_tenA-F Lclaclac_tenA-R	71	DSM 27303 (Formaggella raw milk cheese)	(Dreier et al., 2021)
*Lactobacillus helveticus*	LbhelvF1 (pheS), LbhelvR (pheS),	89	DSM 20075 (emmental cheese)	(Dreier et al., 2021)
*Leuconostoc mesenteroides*	Lnmesen_purL-F Lnmesen_purL-R	92	DSM 20346 (Hansen's dried starter powder)	(Dreier et al., 2021)
*Streptococcus thermophilus*	Sctherm_yeeO-F Sctherm_yeeO-R	72	DSM 20617 (pasteurized milk)	(Dreier et al., 2021)

Note: ^a^ The L. *casei* group includes L. *casei*, *L. paracasei*, *L. rhamnosus*, and *L. zeae* (Furet et al., 2004).

For the primer pairs adopted from Dreier et al. (2021), the qPCR assays were performed in a total reaction mix volume of 12 μL, containing 6 μL 2 × SSoAdvanced Universal SYBR® Green Supermix (Bio Rad, Hercules, USA), 500 nM of forward and reverse primers and 2 μL of DNA (c = 1 ng/μL). The qPCR cycling conditions comprised an initial denaturation at 95 °C for 1 min, followed by 39 cycles of 95 °C for 5 s and 60 °C for 1 min (Dreier et al., 2021). For the primer pair adopted from Furet et al. (2004), a concentration of 900 nM of forward and reverse primers was used. The qPCR conditions for this primer pair were as follows: 50 °C for 2 min, 95 °C for 10 min, and 40 cycles of amplification (95 °C for 15 s, 60 °C for 1 min) (Furet et al., 2004). The melting curve analysis was performed using a gradient from 60 to 95 °C, with 1 °C steps per 3 s. Standard calibration curves were generated using 10-fold serial dilutions of isolated DNAs with a starting quantity of 2 ng (Fig. S2 - Fig. S8). The DNA mass (ng) was determined for each sample for each target using the standard calibration curves and the results were expressed as relative amounts (%). The qPCR data in ng for each measurement are provided in the Supplementary Material, Table S1.

### 2.3 Determination of free amino acid (FAA) content by IEC with post-column NINHYDRIN derivatization

0.20 g of powdered cheese was weighed into a 10 mL tube. Subsequently, 5 mL of 0.2 N tri‑sodium citrate buffer (pH 2.2), consisting of citric acid monohydrate and sodium citrate dihydrate both purchased from Merck, Darmstadt, Germany adjusted with 1 N HCl (Lactan, Graz, Austria) was added to the sample, which was vortexed and placed into a preheated ultrasonic bath at 30 °C for 15 min. The sample was filtered using a ROTILABO® CME 0.45 μm filter (Carl Roth, Karlsruhe, Germany) and transferred into 2 mL tubes (Eppendorf, Hamburg, Germany). Then, 500 μL of the filtered sample was mixed with 1000 μL of lithium citrate loading buffer (pH = 2.2). From this mixture, 500 μL was combined with 50 μL internal standard (S-2-aminoethyl-l-cystein-hydrochloride, 2.5 μmol L^−1^, Merck, Darmstadt, Germany) and 50 μL sulfosalicylic acid (50 g/100 mL, Merck, Darmstadt, Germany). The resulting mixture was vortexed and centrifuged at 680*g* for 5 min using the Mikro 200 Centrifuge (Andreas Hettich, Tuttlingen, Germany). Next, 300 μL of the supernatant was combined with 300 μL of lithium citrate loading buffer and 30 μL of 1 mol L^−1^ sodium hydroxide (Merck, Darmstadt, Germany). Amino acid analysis was conducted by the Biochrom 30+ Physiological High-Performance LC System (Biochrom, Cambridge, UK). The system employed five lithium citrate buffers with pH ranging from pH 2.80 to 3.55 as the mobile phase. The column was regenerated after each run with lithium regeneration buffer having a pH above 13. The mobile phase was delivered at a constant flow rate of 25 mL/h. The flow rate of the ninhydrin solution was set to 20 mL/h. The chromatographic separation was as follows: Initial equilibration was carried out at 32 °C with Buffer 1 for 5 min (split into four short steps of 1, 3, 0, and 1 min), with the ninhydrin reagent switched off. After this, the ninhydrin reagent was activated and the system continued with Buffer 1 at 32 °C for 6.5 min. The separation then proceeded with Buffer 2 at 32 °C for 33 min, followed by Buffer 3 at 45 °C for 13.5 min, and then Buffer 3 at 50 °C for 3.5 min. Subsequently, the temperature was increased to 62 °C and Buffer 4 was used for 21 min. The temperature was raised to 79 °C, with Buffer 5 running for 35 min, followed by Buffer 6 at 79 °C for 6 min, and Buffer 1 at 79 °C for another 6 min, all while the ninhydrin reagent remained active. The detection phase concluded with Buffer 1 at 79 °C for 20 min, during which the ninhydrin reagent was switched off. After detection, a cleaning and re-equilibration phase was performed: the system was flushed with no buffer at 50 °C for 2 min with the pump and ninhydrin reagent off, followed by Buffer 1 at temperatures gradually lowered from 30 to 32 °C over approximately 12 min, with the ninhydrin reagent off. The entire method lasted 163.5 min (2 h and 43.5 min).

The external standard calibration was measured with three different physiological standard mixes containing a total of 41 amino acids with concentrations ranging from 0.525 to 1 μmol × mL^−1^. The physiological standard mixes were purchased from Laborservice Onken (Laborservice Onken, Gründau, Germany). The lithium-containing buffers and ninhydrin were purchased from Biochrom (Biochrom, Berlin, Germany). The amino acids were individually summed, and the results were expressed per 100 g of dry matter. The dry matter of the cheese samples was determined thermogravimetrically by drying the samples to a constant weight at 102 °C, following the method described by Matissek, Steiner, & Fischer (2014). Concentrations of the individual amino acids can be found in the Supplementary Material (Table S4).

### 2.4 Volatile fraction characterization by HS-SPME/GC–MS

HS-SPME/GC–MS was conducted on a TRACE 1300 gas chromatograph (Thermo Fisher Scientific, Milan, Italy) coupled with an ISQ mass spectrometer (Thermo Fisher Scientific, Milan, Italy) equipped with an electronic impact source. The applied method was adapted from Bettera et al. (2023). 2 g of powdered cheese was weighed into a 20 mL vial. Then 5 μL of Toluene were added (100 mg × L^−1^, Sigma, St. Louis, USA), as an internal standard. The equilibration phase of the headspace consisted of 20 min at 40 °C and was followed by the extraction phase at 40 °C for 20 min using a SPME fiber functionalized with a divinylbenzene-carboxen-polydimethylsiloxane coating (Supelco, Bellefonte, USA). Analyte desorption was conducted at 250 °C for 2 min. A Supelcowax 10 capillary column (Supelco; 30 m × 0.25 mm × 0.25 μm) was used to separate the analytes. The starting oven temperature was set at 50 °C and maintained for 3 min. Next, the temperature was increased to 130 °C (5 °C/min), and then to 220 °C (15 °C/min). Then, the temperature was maintained at 220 °C for 10 min. For the detection, the mass spectrometer was set in full scan mode and registered the spectra in the mass range from 35 to 400 *m*/*z*. Peaks were identified by comparing the experimental mass spectra with those reported in the NIST14 instrument library and by the linear retention indexes (LRIs) calculation based on the analysis of a C_8_ - C_20_ alkane standard solution (Sigma-Aldrich, Milan, Italy) under the same conditions. The identified molecules were semi-quantified by comparing the relative peak areas of the identified volatiles with the peak area of Toluene.

### 2.5 Sensory evaluation using the triangle test method

Finally, three triangle tests were conducted according to ISO 4120:2004 (International Standard Organization, 2004) to determine whether differences evidenced by advanced techniques were also perceptible by consumers.

All possible cheese combinations of the sample set were tested (Table 3). The triangle tests were conducted with 24 participants (50 % female) aged between 20 and 59 years, with an average age of 28.8 years. The panelists decided voluntarily and were also informed that smoking, eating, or drinking must be avoided one hour before the sensory test. To obtain a heterogeneous sample, cheese consumers without sensory training from administrative, teaching, research staff, and students were recruited from MCI | The Entrepreneurial School and agreed to be included in the testing.

**Table 3:** Sample combinations for three triangle tests.

Triangle test	Tested samples
1	Tiroler Bergkäse PDO (D_BKGU) - Bergkäse w/o PDO (D_BK)
2	Bergkäse w/o PDO (D_BK) - Stilfser type w/o PDO (D_BATO)
3	Tiroler Bergkäse PDO (D_BKGU) - Stilfser type w/o PDO (D_BATO)

### 2.6 Statistical analysis

A non-parametric one-way ANOVA test (Kruskal-Wallis test with Dunn's multiple comparison posthoc test with Bonferroni correction) was performed in R Studio Version 2024.4.1.748 (Posit team, 2024) to assess significant differences between the three types of cheese for each target species, the sum of FAAs, each chemical class, and each volatile compound. Spearman rank correlation was used to assess the relationship between ripening time and FAA levels. The level of significance was set to α = 0.05. Next, all data obtained from three methods (qPCR, IEC, HS-SPME/GC–MS) were combined and a clustered heatmap was drawn using the Euclidian method and the package “pheatmap”. The orthogonal partial least squares discriminant analysis (OPLS-DA) was performed with combined data using SIMCA® (version 17.0, 2022, Sartorius, Göttingen, Germany). The triangle tests were interpreted according to ISO 4120:2004 (International Standard Organization, 2004). *p* - values were calculated using the package “SensoMineR”. The level of significance was set to α = 0.05.

## 3 Results and discussion

The advantage of investigating real-life, commercially sold cheeses, rather than model cheeses, is that they reflect the natural variability present in actual production, providing valuable insights for the classification of the PDO and non-PDO cheeses under examination. Non-PDO Stilfer cheese has been used as control since it differs substantially from both PDO and non-PDO Bergkäse in terms of milk treatment (pasteurized milk vs. raw milk), physicochemical composition (moisture, texture, fat and salt content), ripening dynamics (shorter vs. longer ripimg times, bacterial composition), and sensory attributes (mild and buttery flavor profile vs. nutty, savoury and more complex). These distinctions support its use as a meaningful internal control in comparative analyses.

### 3.1 Study of the bacterial composition

To confirm sufficient bacterial diversity across cheese types a preliminary Sanger sequencing-based investigation of cloned PCR amplicons representing 16S rDNA of different bacterial genera was conducted. The results are reported in the Supplementary Material, Table S2. The distribution of bacterial genera in ripened cheese deviates from that of starter cultures (Fox, McSweeney, Cogan, & Guinee, 2004) and an additional genus (*Lacticaseibacillus*), introduced by the use of raw milk, was detected. For example, previous studies on molecular profiling of cheeses such as Parmigiano Reggiano (Bertani et al., 2020) and French cheese varieties (Dugat-Bony et al., 2016) have identified specific bacterial taxa, including Lactobacillus, Leuconostoc, and Streptococcus, as dominant during ripening. Similarly, our study found that these genera play a significant role in the microbial composition of Tiroler Bergkäse PDO.

**Fig. 2:**
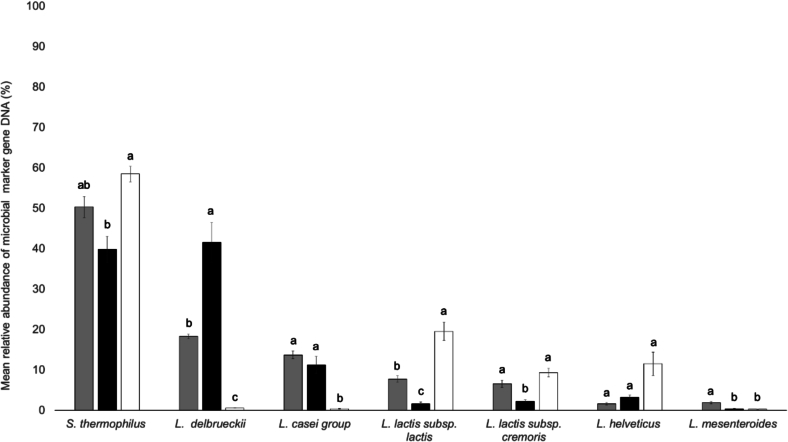
Comparison of the relative abundance of microbial marker gene DNA (%) across cheese types. Bars represent mean values ± standard error. Bergkäse w/o PDO (grey), Tiroler Bergkäse PDO (black), Stilfser type w/o PDO (white). Significant differences were assessed by Kruskal-Wallis test with Dunn's multiple comparison posthoc test with Bonferroni correction. Bars with different letters indicate significant differences. Bars sharing the same letter are not significantly different from each other (α = 0.05).

The bacterial profile within the Bergkäse type is notably more similar compared to the differences observed between the Bergkäse and Stilfser type (Fig. 2). Although the traditional specification of Tiroler Bergkäse PDO does not explicitly mention the type of starter cultures to be used, it was included in the analysis because this study is based on real samples. These include both PDO and non-PDO Bergkäse currently produced in Tyrol, allowing to capture the diversity of actual production practices. As starter cultures can differ between producers and influence the bacterial composition and sensory characteristics of the cheese, they represent an important factor in distinguishing between cheeses. This alignment within the Bergkäse group is reflected in the qPCR results, which largely confirm starter culture compositions while demonstrating their changing proportions. In Bergkäse without PDO, all expected marker gene DNA was detected as anticipated from starter culture composition. While in Tiroler Bergkäse PDO, in which the presence of marker gene DNA from the starter culture was confirmed, small amounts of marker gene DNA of L. *lactis* subsp. *lactis* (1.6 ± 0.4 %) and L. *lactis* subsp. *cremoris* (3.2 ± 0.4 %) were also found (Fig. 2). The presence of marker gene DNA of these microorganisms, which are not part of the cheese's starter culture, could be attributed to their introduction from the cheese-making environment or their presence in the raw milk (Beresford, Fitzsimons, Brennan, & Cogan, 2001; Fernández, Alegría, Delgado, Martín Cruz, & Mayo, 2011). Since the method targets microbial marker gene DNA, the results reflect total DNA presence and do not distinguish between live and dead cells. Thus, DNA from thermosensitive bacteria may still be detected even if the organisms did not survive the cheese production process.

In contrast Stilfser type without PDO only a minimal amount of L. *delbrueckii* marker gene DNA (0.6 ± 0.05 %) was detected (Fig. 2). However, an average of 16.9 ± 1.5 % of *L. helveticus* marker gene DNA was found in the Stilfser type without PDO in two out of three production days, which does not match the starter culture specification. This discrepancy could be due to misinformation or contamination that occurred during those production days. On the last day of production, only 0.6 ± 0.2 % of L. *helveticus* marker gene DNA was detected, which aligns with the information provided by the manufacturer.

Tiroler Bergkäse PDO shows significant differences from Bergkäse without PDO in the relative marker gene DNA abundance of L. *delbrueckii*, *L. lactis* subsp. *lactis*, *L. lactis* subsp. *cremoris*, and L. *mesenteroides* (α = 0.05). Those significant differences between the very similar cheeses can be attributed to the different starter cultures used for their production. The relative abundance of the marker gene DNA of L. *delbrueckii* is highest in Tiroler Bergkäse PDO (41.5 ± 4.9 %) (Fig. 2). This can be explained by the fact that Tiroler Bergkäse PDO uses a starter culture with fewer diverse organisms overall. Many studies have investigated the effects of adding specific microorganisms to the starter culture of cheese and of using a more diverse starter culture (Bassi, Puglisi, & Cocconcelli, 2015). In Caciotta type cheeses, the addition of other lactic acid bacteria, such as *L. curvatus*, alongside *S. thermophilus* and L. *delbrueckii*, has been shown to improve both flavor and texture (Di Cagno et al., 2011). Similarly, the introduction of the non-starter lactic acid bacterium L. *paracasei* has been demonstrated to enhance the taste of Gouda cheese (van Hoorde et al., 2010).

Other research has indicated that the incorporation of certain bacterial strains can reduce the content of biogenic amines (Renes et al., 2014), and certain bacteria can also contribute to the inhibition of *E. coli* (Piras et al., 2013). This means that the use of a wider variety of microorganisms, rather than a limited number, can offer several benefits, including improved organoleptic properties, reduced biogenic amine content, and enhanced inhibition of pathogenic bacteria.

The microorganisms L. *lactis* subsp. *lactis,* and L. *lactis* subsp. *cremoris* are not present in the starter culture of Tiroler Bergkäse PDO and may originate from the environment or the raw milk (Beresford et al., 2001; Fernández et al., 2011). However, their impact on the relative abundance of marker gene DNA differs significantly from the Bergkäse without PDO, where these organisms are directly introduced through the starter culture (α = 0.05). The same applies to L. *mesenteroides*.

The L. *casei* group is the dominant microbial group found in long-ripened raw milk cheeses such as Parmigiano Reggiano (Bettera et al., 2023). Raw milk is the primary source of L. *casei* group members (Bottari, Levante, Neviani, & Gatti, 2018). In Stilfser type cheese without PDO, the microbial marker gene DNA from the L. *casei* group constitutes an average of 0.30 ± 0.05 %. In contrast, this group accounts for 12.50 ± 1.20 % of the microbial marker gene DNA in both Tiroler Bergkäse PDO and Bergkäse, reflecting their status as raw milk cheeses. Significant differences exist between the two Bergkäse types and the Stilfser type cheese regarding the relative abundance of L. *casei* group marker gene DNA derived from raw milk (α = 0.05) (Fig. 2).

Unlike qPCR, which targets predefined sequences, NGS enables the identification of a broad spectrum of microbial species without prior knowledge of their presence (Cao, Fanning, Proos, Jordan, & Srikumar, 2017). This untargeted approach allows the discovery of novel or unexpected microbial taxa and species, which is particularly relevant in complex microbial ecosystems such as cheeses. In this initial study, however, Next-Generation Sequencing (NGS) was not applied. Despite this, the presented study still provided valuable insights into the distribution of starter organisms and the L. *casei* group introduced by raw milk. A limitation of the selected Sanger sequencing compared to NGS is its ability to detect variants only if they represent a larger proportion of the DNA. However, this is only a minor limitation in this study, since it focuses on the main bacterial strains only. It is important to note that the preliminary cloning experiment exclusively detected organisms from the starter culture as well as the raw milk group, with no pathogens identified.

Because qPCR detects total DNA, it cannot distinguish between viable and non-viable bacterial cells. This limitation is particularly relevant in ripened cheeses, where heat-, salt-, or acid-sensitive microorganisms may be inactivated early in the production process but their DNA can persist throughout ripening. As a result, qPCR-based quantification may overestimate the contribution of certain taxa to the mature cheese microbiome, reflecting historical presence rather than current metabolic activity. Consequently, the bacterial composition obtained through qPCR should be interpreted as an indicator of microbial DNA abundance rather than a direct measure of active microbial populations, which may differ substantially during later stages of ripening. Also, temporal limitations reduce the generalizability of these findings, therefore, the inclusion of samples from various seasons might improve the relevance of further studies. However, this will also lead to a higher variability and thus reduce specifity.

In summary, the typicity of each cheese type is reflected in the relative abundance of specific microbial marker gene DNA. Tiroler Bergkäse PDO is characterized by a high proportion of L. *delbrueckii* marker gene DNA and lower diversity of starter organisms overall. In contrast, Bergkäse without PDO shows a broader microbial composition including *Streptococcus thermophilus*, *L. lactis* subspecies, and L. *mesenteroides*. The L. *casei* group, originating mainly from raw milk, is significantly more abundant in both raw milk cheeses Tiroler Bergkäse PDO and Bergkäse compared to Stilfser type cheese. Stilfser type cheese is further distinguished by variable presence of L. *helveticus* and minimal L. *delbrueckii*, reflecting differences in starter cultures and production conditions.

### 3.2 FAA content trend

Although individual results for the free amino acids are available (see Supplementary Material, Table S3), the analysis was deliberately focused on the sum of amino acids. This parameter is both biologically more relevant to the degree of ripening and statistically less susceptible to random fluctuations in individual values. In addition, the sum of single values can explain the consistent and significant differences observed between the cheese types, providing a solid basis for evaluation.

**Fig. 3:**
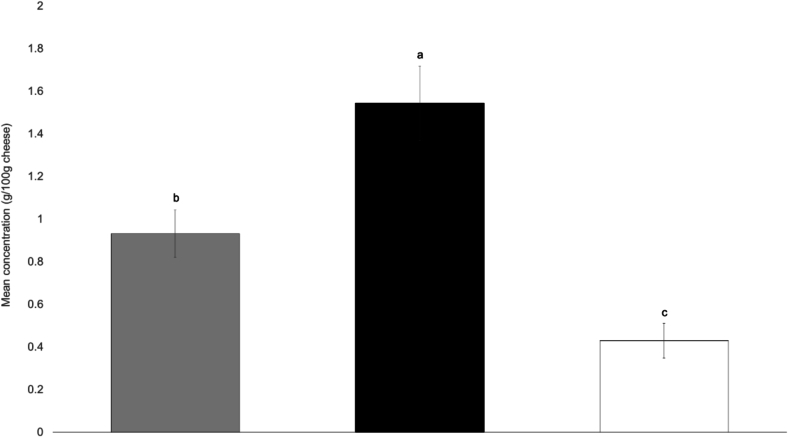
Comparison of the concentration of the total free amino acid content (mg/100 g dry matter) across cheese types. Bars represent mean values ± standard error. Bergkäse w/o PDO (grey), Tiroler Bergkäse PDO (black), Stilfser type w/o PDO (white). Significant differences were assessed by Kruskal-Wallis test with Dunn's multiple comparison posthoc test with Bonferroni correction. Bars with different letters indicate significant differences (α = 0.05).

The FAA content is highest in Tiroler Bergkäse PDO (2335.74 ± 160.55 mg/100 g dry matter), intermediate in Bergkäse without PDO (1434.05 ± 98.44 mg/100 g dry matter), and lowest in Stilfser type without PDO (722.97 ± 80.27 mg/100 g dry matter). Significant differences in the FAA content are observed between all tested cheeses (Fig. 3).

A Spearman correlation test performed between ripening time and total FAA levels, revealed a strong positive relationship (ρ = 0.736, *p* < 0.001), suggesting that extended ripening promotes the accumulation of free amino acids. Mayer and Fiechter (2013) analyzed FAAs as their aminoquinolyl-N-hydroxysuccinimidyl carbamate derivatives using ultra-high performance liquid chromatography in 28 commercial cheese samples, including one unspecified Bergkäse. They measured a total of 2.01 g/100 g of FAAs in this sample (Mayer & Fiechter, 2013). In the present study, the overall average FAA content for Tiroler Bergkäse PDO was found to be 1.54 ± 0.10 g/100 g, expressed relative to the total cheese mass (wet weight). Considering the inherent variability in cheese production, potential methodological differences, as well as the lack of detailed information on the Bergkäse considered in the previous research, the results of the present study are consistent with those presented by Mayer & Fiechter, 2013 (Mayer & Fiechter, 2013).

The results are consistent with existing literature, which indicates that cheeses subjected to longer ripening periods tend to have a higher total FAA content compared to those with shorter ripening times (Abarquero et al., 2024; Niro et al., 2017). Additionally, a previous study has shown that raw milk cheeses (Bergkäse type) exhibit a greater FAA content than those made from pasteurized milk (Stilfser type) (Gaya, Sánchez, Nuñez, & Fernández-García, 2005; Pappa & Sotirakoglou, 2008).

Cheeses made from raw milk typically contain higher levels of free amino acids (FAAs) than those made from pasteurized milk. This can be attributed to several factors. First, raw milk cheeses harbor a more diverse and active non-starter microbial community, which contributes additional proteolytic enzymes beyond those produced by starter cultures. Second, raw milk contains indigenous enzymes (e.g., cathepsin D and plasmin) that remain active during ripening and further contribute to protein breakdown (Gaya et al., 2005).

Furthermore, the literature suggests that cheeses produced with thermophilic cultures (Tiroler Bergkäse PDO) have the highest levels of FAAs, while those made with mixed thermophilic-mesophilic cultures (Bergkäse without PDO) display intermediate levels, and cheeses made with mesophilic cultures (Stilfser type without PDO) contain the lowest FAA content (Pappa & Sotirakoglou, 2008). This because thermophilic bacteria, such as *S. thermophilus*, possess additional peptidases (e.g., PepS, oligopeptidase) and show higher specific activities of PepX, PepN, and PepC compared to mesophilic cultures like L. *lactis*. Moreover, thermophilic cultures have also been shown to release intracellular enzymes earlier during ripening due to earlier cell lysis, thereby enhancing proteolysis (Pappa & Sotirakoglou, 2008).

FAA contents were expressed in relation to the dry matter content of the respective cheese samples. This is because the cheese ripening involves a complex set of biochemical transformations (Fox, Guinee, Cogan, & McSweeney, 2017), affecting the entire composition of the cheese. Free amino acids are the end products of protein breakdown, but their formation is influenced by various factors such as the fat and salt content (McCarthy, Kelly, Wilkinson, & Guinee, 2017). The approach of relating amino acids to the dry matter content rather than the total cheese mass (wet weight) draws a more comprehensive picture of the cheeses, reflecting the interdependent changes in cheese structure and composition throughout the maturation process.

The FAA contents differ between the cheeses, making the parameter ideal for the multivariate analysis. In the multivariate analysis, differences in ripening times were accounted for by separately analyzing the cheeses according to their respective production days.

### 3.3 Volatile fraction evaluation

A total of 22 different volatile compounds were identified in the headspace of the cheeses by using the NIST14 instrument library and the Kovats retention indexes (Kováts, 1958) (Supplementary Material, Table S4). The 22 identified volatile compounds are grouped into five chemical classes, including eight acids (*acetic acid, propanoic acid, isobutyric acid, butyric acid, isovaleric acid, valeric acid, hexanoic acid, octanoic acid*), five alcohols (*ethanol, 3-methyl-1-butanol, 2-heptanol, 1-hexanol, 2,3-butanediol*), three aldehydes (*3-methyl-butanal, hexanal, benzaldehyde*), five ketones (*biacetyl, 2-pentanone, 2-heptanone, acetoin, 2-nonanone*), and one sulfur compound (*dimethyl disulfide*).

The samples are characterized by acids (43.9–90.3 %), alcohols (1.0–36.0 %), ketones (0.9–27.8 %), aldehydes (0.0–6.5 %), and one sulfur compound (0.0–1.3 %) (Supplementary Material, Table S5). Across the cheese types, the predominant acids identified are *butyric acid* (30.8 ± 2.6 %), *acetic acid* (25.3 ± 1.1 %), and *isovaleric acid* (22.3 ± 2.7 %). The primary alcohols detected, across all cheeses, include *ethanol* (70.4 ± 5.3 %), *1-hexanol* (15.5 ± 5.7 %), and *2,3-butanediol* (10.3 ± 1.9 %). Similarly, the most common aldehydes across the cheese samples are *3-methyl-butanal* (31.0 ± 7.4 %), *benzaldehyde* (25.9 ± 8.2 %), and *hexanal* (24.6 ± 6.5 %). The major ketones present in these cheeses are *acetoin* (60.9 ± 4.9 %), *2-heptanone* (20.2 ± 3.6 %), and *2-pentanone* (14.8 ± 1.8 %). *Dimethyl disulfide* was the only sulfur compound detected and quantified only in Tiroler Bergkäse PDO (Supplementary Material, Table S5).

A significant difference can be observed in the total volatile compound and acid amounts between Bergkäse without PDO and Stilfser type without PDO (α = 0.05). Both Bergkäse type cheeses differ significantly from the Stilfser type cheese in terms of alcohol content (α = 0.05). However, no significant differences were found among the tested cheeses in the chemical classes of aldehydes and ketones (α = 0.05). The sulfur compound, *dimethyl disulfide* was detected only in Tiroler Bergkäse PDO (Table 4).

**Table 4:** Concentrations of total volatiles, acids, alcohols, aldehydes, ketones, and sulfur compound (ng/g). Mean values ± standard error. Significant differences were assessed by Kruskal-Wallis test with Dunn's multiple comparison posthoc test with Bonferroni correction. Different letters indicate significant differences (α = 0.05).

Cheese	Total	Acids	Alcohols	Aldehydes	Ketones	Sulfur compound
Bergkäse w/o PDO	2820.8 ± 272.9^b^	2231.6 ± 167.0^b^	148.5 ± 35.1^b^	50.8 ± 11.3^a^	390.0 ± 106.1^a^	n.d.^b^
Tiroler Bergkäse PDO	3751.1 ± 385.7^a,b^	2774.5 ± 362.6^a,b^	295.2 ± 163.3^b^	128.9 ± 22.0^a^	531.5 ± 60.0^a^	20.9 ± 9.5^a^
Stilfser type w/o PDO	4098.2 ± 258.3^a^	3297.8 ± 302.9^a^	459.4 36.1^a^	56.1 ± 23.4^a^	284.8 ± 82.8^a^	n.d.^b^

Note: n.d. means not detected.

During cheese ripening, various biochemical processes such as lactose and citrate metabolism, lipolysis, and proteolysis lead to the formation of a broad range of potentially flavor-active compounds. These compounds originate from microbial fermentation and enzymatic activity (Fox et al., 2017), and some of them differ significantly between the examined cheese types.

**Fig. 4:**
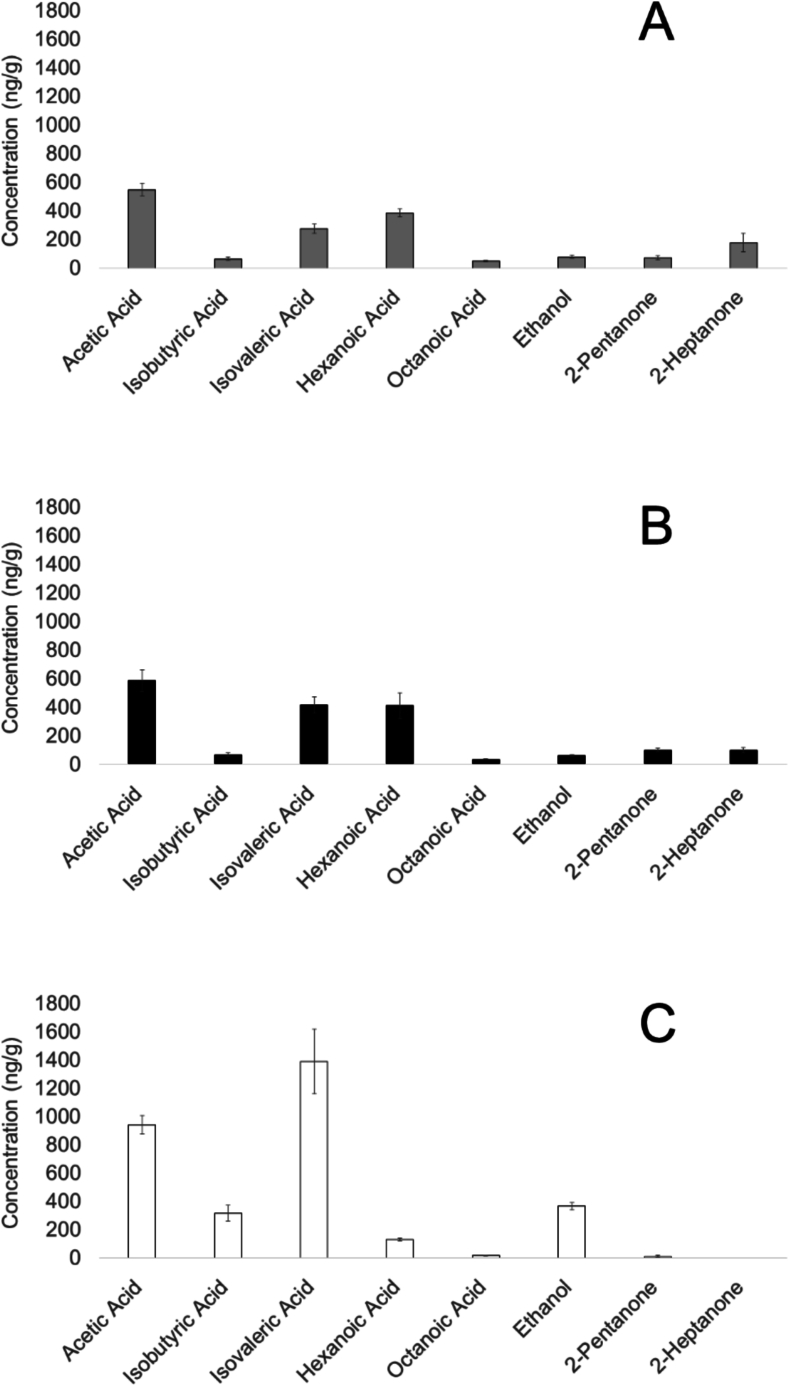
Concentrations of volatiles (ng/g) with statistically significant differences between Bergkäse type cheeses and Stilfser type w/o PDO. Bars represent mean values ± standard error. Bergkäse w/o PDO (A, grey), Tiroler Bergkäse PDO (B, black), and Stilfser type cheese (C, white). Statistical differences were determined by Kruskal-Wallis test with Dunn's multiple comparison posthoc test with Bonferroni correction (α = 0.05).

In total, eight compounds can statistically discriminate between Bergkäse and Stilfser cheese including *acetic acid*, *isobutyric acid*, *isovaleric acid*, *hexanoic acid*, *octanoic acid, ethanol*, *2-pentanone,* and *2-heptanone* (α = 0.05, Fig. 4). Among the organic acids and alcohols, compounds like acetic acid and ethanol are produced primarily by heterofermentative lactic acid bacteria including *Leuconostoc*, *Oenococcus*, *Weissella*, and certain *Lactobacillus* species through the phosphoketolase pathway and through lactose and citrate metabolism (Silva et al., 2023). In contrast, branched-chain fatty acids such as isobutyric acid and the isovaleric acid are formed through the catabolism of the amino acids valine, leucine, and isoleucine by bacteria (Yvon & Rijnen, 2001). Straight-chain fatty acids including hexanoic acid and octanoic acid originate mainly from enzymatic lipolysis of milk fat (Fox et al., 2017).

**Fig. 5:**
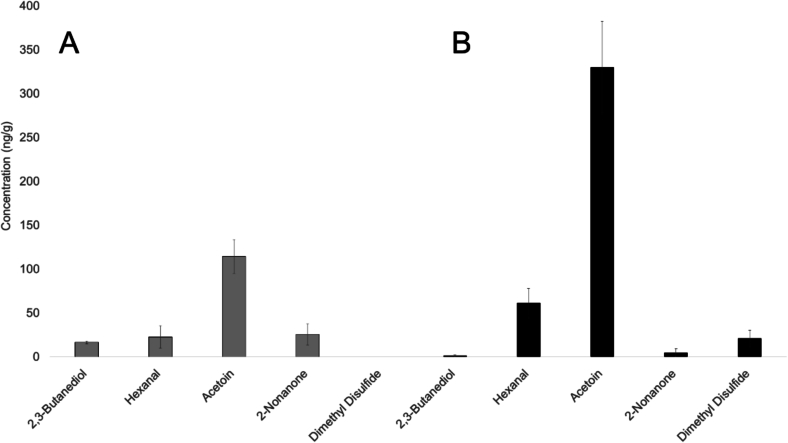
Concentrations of volatiles (ng/g) with statistically significant differences between Tiroler Bergkäse PDO and Bergkäse w/o PDO. Bars represent mean values ± standard error. Bergkäse w/o PDO (A, grey) and Tiroler Bergkäse PDO (B, black). Statistical differences were determined by Kruskal-Wallis test with Dunn's multiple comparison posthoc test with Bonferroni correction (α = 0.05).

Notably, five compounds show significant differences between Tiroler Bergkäse PDO and Bergkäse without PDO including *2,3-butanediol*, *hexanal*, *acetoin, 2-nonanone,* and *dimethyl disulfide* (α = 0.05, Fig. 5). Volatiles like methyl ketones (e.g. 2-nonanone) are also associated with lipid metabolism and reflect differences in microbial activity and ripening conditions (Fox et al., 2017).

Several other PDO cheeses have already been extensively described in the scientific literature using multimethodological approaches. Parmigiano Reggiano (PR) PDO is one of the most thoroughly investigated PDO cheeses. PR has been the subject of numerous analytical studies aiming to characterize its compositional and quality attributes. Advanced techniques such as near-infrared spectroscopy (NIRS), nuclear magnetic resonance (NMR), and ultra-high-performance liquid chromatography (UHPLC) have been applied to assess ripening time, the impact of different feeding systems, and the detection of potential markers of adulteration (Becchi, Rocchetti, Vezzulli, Lambri, & Lucini, 2023; Cavallini et al., 2023; Cevoli et al., 2013; Rocchetti, Michelini, Pizzamiglio, Masoero, & Lucini, 2021; Stocco et al., 2025). In addition, microbial dynamics of PR have also been investigated (Becchi et al., 2025; Gatti, Bottari, Lazzi, Neviani, & Mucchetti, 2014; Levante et al., 2021; Sola et al., 2022). Other cheeses that have been extensively studied include Mozzarella di Bufala Campana PDO (Levante et al., 2023; Romano, Giordano, Chianese, Addeo, & Musso, 2011), Grana Padano PDO, Emmental PDO, and Serra da Estrela PDO (Cardin et al., 2022).

This study focuses on Tiroler Bergkäse PDO due to its significant role in terms of history, tradition, culture, food production, and consumption in Tyrol. Despite its importance, little research has been conducted so far on its characterization. In a 2014 study Huck-Pezzei et al. for example investigated the typicality of alpine foods using near-infrared (NIR) spectroscopy (Huck-Pezzei et al., 2014). Investigating the parameters that characterize the traditional production method of Tiroler Bergkäse PDO is vital for maintaining its distinctiveness and protecting the PDO label from fraud. These parameters include the exclusive use of Tyrolean partially skimmed alpine or valley milk, from cows fed without silage, the use of calf rennet, the heating of the curd to approximately 52 °C, the pressing and salting in a 20 % sodium chloride brine, the aging at 12–16 °C at 90–95 % humidity, and regular brushing with brine twice a week. Furthermore, no antioxidants, preservatives, or colorants are added, and the cheese is considered ready for consumption after a minimum aging period of three months (Tiroler Milchkäuferverband, 1997). According to the traditional specification, no explicit details are given regarding the type of starter cultures to be used. According to Codex Alimentarius Austriacus, the cheese is considered ready for consumption after at least three months of aging (Bundesministerium für Soziales, Gesundheit, Pflege und Konsumentenschutz (Ed.), 2024). Finally, the cheese exhibits a brown-yellow to brown rind, weighs at least 12 kg, has a firm to smooth texture, an ivory to pale yellow interior, and offers a mild to slightly piquant flavor (Tiroler Milchkäuferverband, 1997).

Various parameters have been proposed in the literature for characterizing PDO cheeses. The microbiota of cheeses, for example, is closely linked to the manufacturing process and can therefore be used to discriminate between them (Cardin et al., 2022). During ripening, proteins are broken down into free amino acids (FAAs) by proteolytic enzymes, increasing their concentration over time (Moreira et al., 2018; Niro et al., 2017; Pappa & Sotirakoglou, 2008). Literature also reports variations in the total FAA content based on the type of starter culture used (mesophilic, mixed thermophilic-mesophilic, thermophilic) (Pappa & Sotirakoglou, 2008). Therefore, it can be assumed that analyzing free amino acids is also useful for distinguishing manufacturing processes, such as those differing in maturation time or the use of specific starter cultures. The microbiota's metabolic activities produce volatile compounds such as aldehydes, ketones, and free fatty acids, which contribute to the cheese's aroma. The FAA profile, which is largely shaped by the microbiota and the ripening time (You et al. 2024), is linked to the volatile fraction of cheese, as these FAAs serve as precursors to aroma compounds (Pappa & Sotirakoglou, 2008). These volatiles, influenced by the microbiota and ripening, changes related to the maturation process and microbiological composition (Cardin et al., 2022; Sánchez-Palomo, Díaz-Maroto, & Pérez-Coello, 2005).

### 3.4 Multivariate analysis

To strengthen the previous findings and correlate the parameters measured by the three different methods, a multivariate analysis was performed. Therefore, data were combined and the cheese samples were clustered based on the volatile profile, the bacterial composition, and the sum of FAAs, using the Euclidian method as well as OPLS-DA.

The differences in the ripening time within each cheese type were relatively small. For Bergkäse without PDO, the variation corresponds to a maximum difference of five days. Tiroler Bergkäse PDO showed a slightly larger range, with a maximum difference of six days. In contrast, Stilfser type without PDO had a wider range, leading to a maximum difference of nine days. However, the overall ripening differences were more pronounced between cheese types, with Stilfser type without PDO having a shorter maturation period due to its general production characteristics. For Bergkäse without PDO and Tiroler Bergkäse PDO, it was not possible to obtain samples with identical ripening times. However, this variation was accounted for in the multivariate analysis, where each wheel from each production day was analyzed separately and the ripening time (days) was included as an additional parameter.

**Fig. 6:**
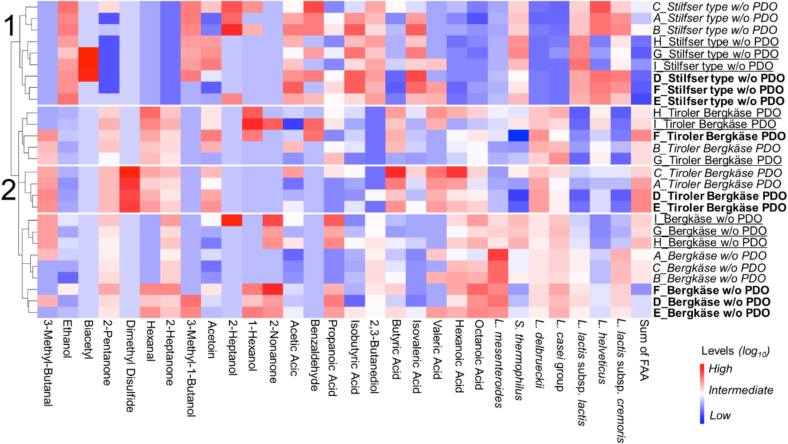
Cheese samples clustering (vertical axis) based on volatile compounds, bacterial profile, and FAA content (horizontal axis) using the Euclidian method. The content or relative abundance increases from blue to red. Samples in italics correspond to the first production day, samples in bold correspond to the second production day, and samples with an underline correspond to the third production day. (For interpretation of the references to colour in this figure legend, the reader is referred to the web version of this article.)

Notably, the two Bergkäse-type cheeses show a high degree of similarity, forming one main cluster (categorized as 2), while the Stilfser type (categorized as 1) stands apart (Fig. 6). This clustering pattern mirrors previous findings from bacterial and volatile profiles, reinforcing the distinction between the two cheese types (Fig. 6). Interestingly, among the non-PDO cheeses, samples from each production day align perfectly within the same subclusters. However, for the Tiroler Bergkäse PDO, produced by a smaller manufacturer, there is a noticeable variation. For instance, the sample G_Tiroler Bergkäse PDO (G_BKGU) (produced on December 2, 2022) clusters more closely with B_Tiroler Bergkäse PDO (B_BKGU) (produced on October 1, 2022) rather than with H_Tiroler Bergkäse PDO (H_BKGU) or I_Tiroler Bergkäse PDO (I_BKGU), despite their shared production day. This indicates potential variability in the PDO production process likely attributable to fluctuations in the concentration of dimethyl disulfide (Fig. 6). The variability observed in these samples is a crucial factor when considering the identification of authenticity. Such indications must only exhibit limited variation to be reliable. If an indicator shows too much fluctuation, it may lose its effectiveness for distinguishing authentic products (Bayen et al., 2024).

A moderate negative correlation between the quantity of L. *helveticus* marker gene DNA and the acetoin content in Stilfser type cheese can be observed (Pearson correlation coefficient = −0.727, *p* = 0.03). This is specifically important as during the first two production days, an average of 16.9 ± 1.5 % *L. helveticus* marker gene DNA was detected, which did not align with the starter culture specification. On the third day, however, only 0.6 ± 0.2 % was detected, which was consistent with the provided information. This variation in L. *helveticus* marker gene DNA appears to influence the *acetoin* content, with higher L. *helveticus* levels correlating with lower *acetoin* concentrations (117.64 ± 44.6 ng/g vs. 506.2 ± 50.9 ng/g) (Fig. 6). This inverse relationship between L. *helveticus* and acetoin is in line with previous studies (Sıçramaz, Güven, Can, Ayar, & Gül, 2022).

The levels of *2,3-butanediol* are lower in Tiroler Bergkäse PDO compared to Bergkäse without PDO. Since *acetoin* can be derived from the citrate metabolic pathway and is converted into *2,3-butanediol* by the enzyme 2,3-butanediol dehydrogenase (Eramo et al., 2024), this may explain why *acetoin* concentrations are higher in Tiroler Bergkäse PDO, while *2,3-butanediol* levels remain lower. This observation appears also to be linked to the relative abundance of L. *lactis* subsp. *lactis* marker gene DNA, which shows a significant difference between the PDO and Bergkäse without PDO cheese (α = 0.05). Given that L. *lactis* subsp. *lactis* is known to produce *2,3-butanediol* via its *2,3-butanediol* dehydrogenase (Crow, 1990), these variations in microbial composition may help explain the differences in *2,3-butanediol* content between the two types of cheese (Fig. 6).

*Hexanal* is produced through lipid peroxidation (from the β-oxidation of linoleic and linolenic acids) (Masotti, Battelli, & De Noni, 2012). It can be viewed as a peroxidation marker (Nzekoue et al., 2019; Panseri, Soncin, Chiesa, & Biondi, 2011). *Hexanal* was found to be significantly different in Tiroler Bergkäse PDO compared to Bergkäse without PDO with higher levels in the PDO cheese. The literature indicates that *hexanal* concentrations increase over time (Nzekoue et al., 2019). Among the tested samples, Tiroler Bergkäse PDO had the longest ripening period.

**Fig. 7:**
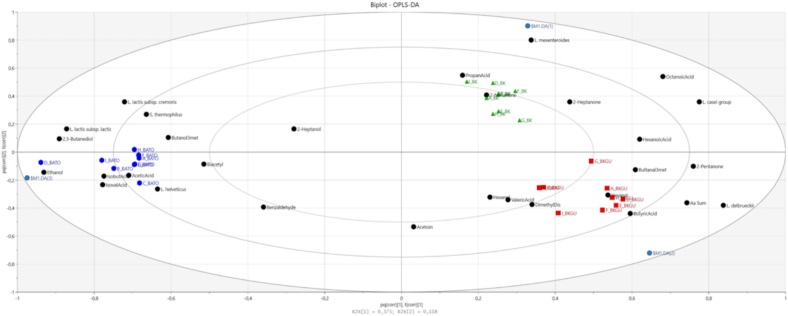
Biplot OPLS-DA of Stilfser type without PDO (BATO, blue), Bergkäse without PDO (BK, green), and Tiroler Bergkäse PDO (BKGU, red). The samples show a separation according to the analyzed variables. Numbers (1−30) indicate the variables: 1 = 2,3-Butanediol, 2 = *L. lactis* subsp. *lactis*, 3 = *L. lactis* subsp. *cremoris,* 4 = *S. thermophilus*, 5 = 3-Methyl-1-Butanol, 6 = Ethanol, 7 = Isobutyric Acid, 8 = Isovaleric Acid, 9 = Acetic Acid, 10 = *L. helveticus*, 11 = Biacetyl, 12 = Benzaldehyde, 13 = 2-Heptanol, 14 = Acetoin, 15 = 1-Hexanol, 16 = Valeric Acid, 17 = Dimethyl Disulfide, 18 = Butyric Acid, 19 = Hexanal, 20 = Sum of FAA, 21 = *L. delbrueckii*, 22 = 2-Pentanone, 23 = 3-Methyl-Butanal, 24 = Hexanoic Acid, 25 = 2-Heptanone, 26 = 2-Nonanone, 27 = Propanoic Acid, 28 = *L. mesenteroides*, 29 = Octanoic Acid, 30 = *L. casei* group. (For interpretation of the references to colour in this figure legend, the reader is referred to the web version of this article.)

The close relationship between the two Bergkäse types is evident, confirming previous findings. Fig. 7 shows the association between *2,3-butanediol* and L. *lactis* subsp. *lactis* as it is known that L. *lactis* subsp. *lactis* produces *2,3 butanediol* via its 2,3-butanediol dehydrogenase (Crow, 1990). Additionally, the non-starter L. *casei* group is clearly associated with the raw milk cheeses. Notably, *dimethyl disulfide*, the total sum of FAAs, *hexanal*, and L. *delbrueckii* show a strong correlation with Tiroler Bergkäse PDO (Fig. 7).

### 3.5 Perceived difference

Three triangle tests were conducted with 24 assessors to evaluate whether the cheese samples were also differently perceived by human assessors. The results show a significant difference for all tested combinations (α = 0.05). The perceived difference between the cheeses belonging to the Bergkäse type is less pronounced than the difference between the Bergkäse type and the Stilfser type cheese, which is consistent with the earlier findings of this study. The multivariate analysis revealed clear associations between certain volatile compounds and the respective cheese varieties. These associations are consistent with the sensory differentiation observed in the triangle tests. For the combination of Bergkäse without PDO and Stilfser type cheese, as well as for the combination Tiroler Bergkäse PDO and Stilfser type, 22 and 23 out of 24 testers, respectively, gave the correct answer. In contrast, for the combination Tiroler Bergkäse PDO and Bergkäse without PDO, 15 out of 24 testers indicated the correct answer. The triangle test is generally a suitable method for detecting sensory differences (Busch-Stockfisch, 2015). However, for a more detailed characterization, it would be preferable to conduct a more comprehensive evaluation with trained panelists, such as using Quantitative Descriptive Analysis (QDA). In this approach, consumers are trained to identify and rate specific sensory attributes that are relevant to the products they are familiar with, making it a bit more accessible than using highly specialized panelists (Busch-Stockfisch, 2015).

## 4 Conclusion

Using qPCR, IEC, and SPME-HS/GC–MS, significant differences were observed between the cheese types (Stilfser and Bergkäse type), as well as between Tiroler Bergkäse PDO and Bergkäse without PDO (α = 0.05). Combining the results from all techniques provided a clearer distinction, allowing for effective separation of the Bergkäse without PDO from Tiroler Bergkäse PDO through cluster analysis and OPLS-DA. A triangle test conducted by human assessors confirmed a significant perceived difference between all tested cheeses (α = 0.05).

The results show that Tiroler Bergkäse PDO, while related to Bergkäse without PDO, exhibits key differences (α = 0.05). Low levels of L. *mesenteroides*, *L. lactis* subsp. *lactis*, and *L. lactis* subsp. *cremoris* marker gene DNA were detected, alongside medium to low levels of *2,3-butanediol* and *2-nonanone*. In contrast, high levels of L. *delbrueckii, hexanal*, *acetoin*, and total FAA were characteristic of the PDO cheese, with statistically significant differences between the PDO and Bergkäse without PDO cheese. Notably, *dimethyl disulfide*, was detected only in four PDO cheese samples and was also significantly different between the two groups showing the high variation in its production compared to the other cheeses investigated (α = 0.05).

This study has demonstrated the potential to distinguish between PDO and non-PDO cheeses using a combination of sophisticated methods, even when the cheeses are produced in close geographical proximity using the same hay milk.

Future studies should aim to analyze a broader range of samples, collected from multiple producers over an extended period, ideally spanning an entire year. This would allow for a more comprehensive understanding of the full variability of the cheese, accounting for seasonal fluctuations in milk composition, production practices, and environmental factors that may influence the final product. By incorporating samples from a wider variety of producers, future studies could capture the diversity of production methods and regional differences, providing a more complete picture of the factors that contribute to the unique characteristics of the cheese. This approach would enhance the robustness of the characterization.

In addition, more detailed and systematic sensory analyses should be conducted to better understand how variations in production manifest in the flavor, texture, and in the overall sensory profile of the cheese. Such analyses would complement the presented data and provide a deeper insight into this traditional food product.

## CRediT authorship contribution statement

**Hannah Innerbichler:** Writing – review & editing, Writing – original draft, Visualization, Methodology, Investigation, Funding acquisition, Formal analysis, Data curation. **Alexander Trockenbacher:** Writing – review & editing, Methodology. **Alexander Höller:** Methodology, Conceptualization. **Sabine Scholl-Bürgi:** Writing – review & editing, Methodology. **Lorenzo Del Vecchio:** Writing – review & editing, Investigation, Formal analysis. **Martina Cirlini:** Writing – review & editing, Methodology, Investigation, Formal analysis. **Jürgen König:** Writing – review & editing, Conceptualization. **Katrin Bach:** Writing – review & editing, Supervision, Resources, Project administration, Methodology, Investigation, Funding acquisition, Data curation, Conceptualization.

## Ethical statement

Participants provided informed consent through the statement “Your answers are confidential and will be evaluated together with those of other participants” where an affirmative reply was required to enter the survey. Participants had also to give the consent for the processing of personal data in accordance with Article 13 of Regulation (EU) 679/2016. They were able to withdraw from the survey at any time without giving a reason. The product tested was safe for consumption.

## Funding

This research was funded by MCI Internationale Hochschule GmbH. This work was supported by Land Tirol [grant number: F.45218/8–2022].

## Declaration of competing interest

The authors declare the following financial interests/personal relationships which may be considered as potential competing interests: Katrin Bach reports financial support was provided by MCI. If there are other authors, they declare that they have no known competing financial interests or personal relationships that could have appeared to influence the work reported in this paper.

## Data Availability

Data will be made available on request.

## Appendix A Supplementary data

Supplementary material Image 1
