# Supplementary material for: Characterization of Tiroler Bergkäse PDO cheese: A multimethodological approach
Source: Food Chem (Oxf). 2025 Dec 8;12:100336. doi: 10.1016/j.fochms.2025.100336 (PMC12807818; doi:10.1016/j.fochms.2025.100336)
Supplement: Supplementary file 1 — Supplementary material [file mmc1.docx]

**Table S1: Supporting sample information.**

| **Sample** | **Date of manufacture** | **Days of ripening** | **Milk type** | **Rennet type** | **Starter culture: composition (%)** |
| --- | --- | --- | --- | --- | --- |
| **Bergkäse w/o PDO** |  |  |  |  |  |
| A-C /BK | 15 October 2022 | 107 | Tyrolean  raw-hay milk | microbial | ***** |
| D-F /BK | 22 November 2022 | 107 |  |  |  |
| G-I /BK | 16 December 2022 | 112 |  |  |  |
| **Tiroler Bergkäse PDO** |  |  |  |  |  |
| A-C /BKGU | 01 October 2022 | 119 | Tyrolean  raw-hay milk | calf | ***** |
| D-F /BKGU | 31 October 2022 | 123 |  |  |  |
| G-I /BKGU | 02 December 2022 | 125 |  |  |  |
| **Stilfser type w/o PDO** |  |  |  |  |  |
| A-C /BATO | 24 November 2022 | 59 | South Tyrolean pasteurized-hay milk | microbial | ***** |
| D-F /BATO | 20 December 2022 | 68 |  |  |  |
| G-I /BATO | 09 February 2023 | 64 |  |  |  |

**Footnotes**

***: on request**


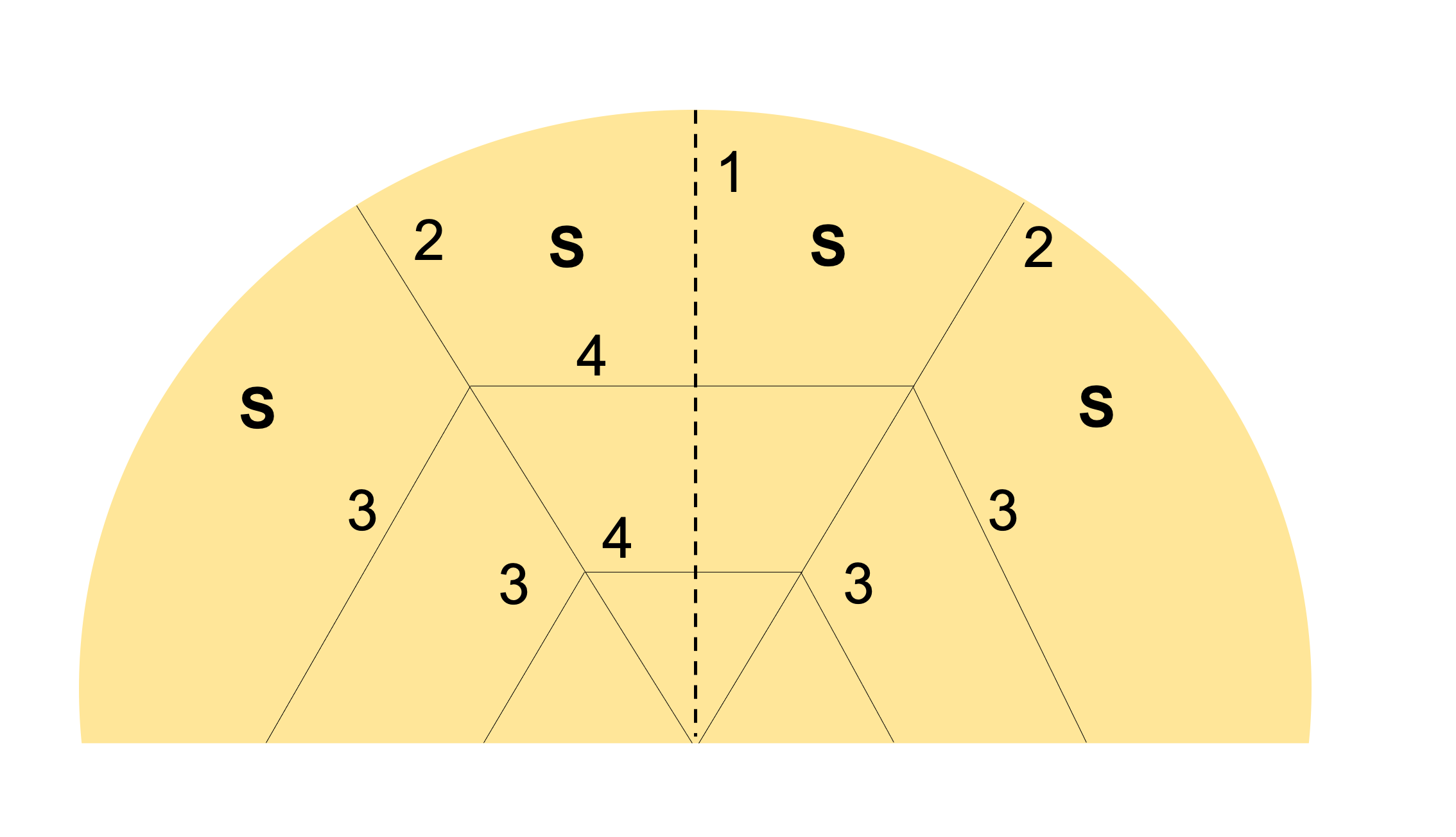


**Figure S1: Sample preparation procedure for cheese subsampling.** 1: Wheels were received as half-loaves, each half-loaf was halved again by drawing a straight line across its middle; 2: Two lines at a 60° angle were drawn from the central axis; 3: Wheels were divided into 6 sections and lines were extended to line 2, respectively; 4: Lines 3 were connected. From the twelve resulting segments, the four outermost pieces were selected for sampling. Additionally, a 2 cm layer from the outer rind was removed before storing the samples.

**Figure S2: Standard curve *L. mesenteroides.***

**Figure S3: Standard curve *S. thermophilus.***

**Figure S4: Standard curve *L. delbrueckii.***

**Figure S5: Standard curve *L. casei* group.**

**Figure S6: Standard curve *L. lactis subsp. cremoris.***

**Figure S7: Standard curve *L. helveticus.***

**Figure S8: Standard curve *L. lactis subsp. cremoris.***

**Table S2: qPCR-based quantification of target species across cheese types (mean ± standard error) expressed in ng.**

| **Bergkäse without PDO** | | | | | | | | | |
| --- | --- | --- | --- | --- | --- | --- | --- | --- | --- |
| **Target** | A_BK | B_BK | C_BK | D_BK | E_BK | F_BK | G_BK | H_BK | I_BK |
| *L. mesenteroides* | 0.059 ± 0.000 | 0.048 ± 0.001 | 0.048 ± 0.001 | 0.043 ± 0.001 | 0.036 ± 0.001 | 0.039 ± 0.001 | 0.022 ± 0.001 | 0.020 ± 0.001 | 0.022 ± 0.000 |
| *S. thermophilus* | 0.965 ± 0.003 | 0.954 ± 0.003 | 1.022 ± 0.007 | 0.834 ± 0.014 | 0.933 ± 0.016 | 0.767 ± 0.011 | 1.167 ± 0.010 | 1.221 ± 0.003 | 1.188 ± 0.013 |
| *L. delbrueckii* | 0.346 ± 0.006 | 0.387 ± 0.006 | 0.366 ± 0.004 | 0.416 ± 0.006 | 0.319 ± 0.002 | 0.407 ± 0.009 | 0.361 ± 0.012 | 0.320 ± 0.004 | 0.361 ± 0.006 |
| *L. casei* group | 0.224 ± 0.006 | 0.231 ± 0.002 | 0.201 ± 0.003 | 0.283 ± 0.004 | 0.342 ± 0.009 | 0.378 ± 0.009 | 0.290 ± 0.008 | 0.254 ± 0.002 | 0.264 ± 0.009 |
| *L. lactis* subsp. *lactis* | 0.178 ± 0.004 | 0.172 ± 0.002 | 0.161 ± 0.000 | 0.216 ± 0.004 | 0.182 ± 0.004 | 0.200 ± 0.004 | 0.091 ± 0.001 | 0.105 ± 0.001 | 0.089 ± 0.003 |
| *L. helveticus* | 0.039 ± 0.000 | 0.029 ± 0.002 | 0.029 ± 0.001 | 0.053 ± 0.001 | 0.051 ± 0.001 | 0.056 ± 0.002 | 0.011 ± 0.001 | 0.013 ± 0.001 | 0.012 ± 0.001 |
| *L. lactis* subsp. *cremoris* | 0.188 ± 0.002 | 0.178 ± 0.001 | 0.173 ± 0.002 | 0.156 ± 0.004 | 0.137 ± 0.000 | 0.154 ± 0.001 | 0.058 ± 0.000 | 0.067 ± 0.000 | 0.064 ± 0.001 |

| **Tiroler Bergkäse PDO** | | | | | | | | | |
| --- | --- | --- | --- | --- | --- | --- | --- | --- | --- |
| **Target** | A_BKGU | B_BKGU | C_BKGU | D_BKGU | E_BKGU | F_BKGU | G_BKGU | H_BKGU | I_BKGU |
| *L. mesenteroides* | 0.005 ± 0.000 | 0.004 ± 0.000 | 0.004 ± 0.000 | 0.007 ± 0.000 | 0.007 ± 0.000 | 0.011 ± 0.001 | 0.006 ± 0.000 | 0.013 ± 0.001 | 0.011 ± 0.000 |
| *S. thermophilus* | 0.765 ± 0.015 | 0.746 ± 0.020 | 1.027 ± 0.026 | 0.604 ± 0.037 | 0.650 ± 0.004 | 0.504 ± 0.006 | 0.863 ± 0.007 | 0.941 ± 0.014 | 1.068 ± 0.014 |
| *L. delbrueckii* | 0.980 ± 0.012 | 0.804 ± 0.043 | 0.549 ± 0.012 | 1.146 ± 0.050 | 1.100 ± 0.001 | 1.250 ± 0.010 | 0.490 ± 0.010 | 0.591 ± 0.007 | 0.564 ± 0.008 |
| *L. casei* group | 0.109 ± 0.001 | 0.273 ± 0.019 | 0.189 ± 0.008 | 0.154 ± 0.007 | 0.141 ± 0.004 | 0.130 ± 0.007 | 0.505 ± 0.006 | 0.329 ± 0.009 | 0.191 ± 0.004 |
| *L. lactis* subsp. *lactis* | 0.050 ± 0.002 | 0.044 ± 0.000 | 0.085 ± 0.002 | 0.015 ± 0.001 | 0.022 ± 0.000 | 0.028 ± 0.001 | 0.016 ± 0.001 | 0.015 ± 0.000 | 0.018 ± 0.000 |
| *L. helveticus* | 0.031 ± 0.001 | 0.067 ± 0.002 | 0.056 ± 0.002 | 0.047 ± 0.003 | 0.047 ± 0.001 | 0.039 ± 0.000 | 0.090 ± 0.002 | 0.087 ± 0.003 | 0.116 ± 0.001 |
| *L. lactis* subsp. *cremoris* | 0.061 ± 0.001 | 0.063 ± 0.003 | 0.089 ± 0.004 | 0.027 ± 0.002 | 0.033 ± 0.001 | 0.038 ± 0.002 | 0.030 ± 0.001 | 0.024 ± 0.000 | 0.033 ± 0.001 |

**Table S2 continued.**

| **Stilfser type without PDO** | | | | | | | | | |
| --- | --- | --- | --- | --- | --- | --- | --- | --- | --- |
| **Target** | A_BATO | B_BATO | C_BATO | D_BATO | E_BATO | F_BATO | G_BATO | H_BATO | I_BATO |
| *L. mesenteroides* | 0.006 ± 0.000 | 0.004 ± 0.000 | 0.005 ± 0.000 | 0.007 ± 0.000 | 0.005 ± 0.000 | 0.007 ± 0.000 | 0.005 ± 0.000 | 0.006 ± 0.000 | 0.006 ± 0.000 |
| *S. thermophilus* | 1.165 ± 0.008 | 1.130 ± 0.011 | 1.109 ± 0.013 | 0.998 ± 0.018 | 1.062 ± 0.011 | 1.187 ± 0.009 | 1.339 ± 0.010 | 1.334 ± 0.008 | 1.195 ± 0.006 |
| *L. delbrueckii* | 0.006 ± 0.001 | 0.014 ± 0.001 | 0.009 ± 0.001 | 0.014 ± 0.001 | 0.014 ± 0.001 | 0.013 ± 0.001 | 0.012 ± 0.001 | 0.012 ± 0.001 | 0.009 ± 0.000 |
| *L. casei* group | 0.004 ± 0.000 | 0.005 ± 0.000 | 0.003 ± 0.000 | 0.006 ± 0.000 | 0.007 ± 0.000 | 0.006 ± 0.000 | 0.018 ± 0.001 | 0.007 ± 0.000 | 0.007 ± 0.000 |
| *L. lactis* subsp. *lactis* | 0.266 ± 0.004 | 0.260 ± 0.004 | 0.248 ± 0.006 | 0.365 ± 0.007 | 0.374 ± 0.000 | 0.351 ± 0.005 | 0.498 ± 0.011 | 0.505 ± 0.009 | 0.652 ± 0.005 |
| *L. helveticus* | 0.323 ± 0.008 | 0.331 ± 0.002 | 0.475 ± 0.009 | 0.339 ± 0.008 | 0.297 ± 0.007 | 0.267 ± 0.005 | 0.019 ± 0.001 | 0.008 ± 0.001 | 0.010 ± 0.000 |
| *L. lactis* subsp. *cremoris* | 0.231 ± 0.002 | 0.256 ± 0.008 | 0.151 ± 0.004 | 0.271 ± 0.003 | 0.241 ± 0.003 | 0.169 ± 0.001 | 0.109 ± 0.001 | 0.127 ± 0.003 | 0.123 ± 0.003 |

**Table S3: Summary of 16S rDNA BLAST analysis representing genera distribution across cheese types by Sanger sequencing of cloned amplicons.**

|  | **N° of clones** | | |
| --- | --- | --- | --- |
|  | Bergkäse without PDO | Tiroler Bergkäse PDO | Stilfser type without PDO |
| BLAST Output | G_BK | G_BKGU | G_BATO |
| *Streptococcus* | 15 | 4 | 8 |
| *Lactobacillus* | 2 | 7 | 0 |
| *Lacticaseibacillus* | 2 | 3 | 0 |
| *Lactococcus* | 0 | 0 | 6 |

**Table S4: Quantification of free amino acids identified by IEC across cheese types (mean ± standard error) in mg/100 g dry matter.**

| **Bergkäse without PDO** | | | | | | | | | | |
| --- | --- | --- | --- | --- | --- | --- | --- | --- | --- | --- |
| **N°** | **FAA** | A_BK | B_BK | C_BK | D_BK | E_BK | F_BK | G_BK | H_BK | I_BK |
|  | **Phosphorylated AA** |  |  |  |  |  |  |  |  |  |
| 1 | Phospho-serine | 28.87 ± 0.94 | 33.3 ± 0.45 | 35.78 ± 1.47 | 31.03 ± 0.77 | 26.17 ± 0.91 | 15.62 ± 0.74 | 42.31 ± 0.42 | 37.22 ± 2.76 | 33.8 ± 1.06 |
|  | **TOTAL** |  |  |  |  |  |  |  |  |  |
|  | **Acidic AA** |  |  |  |  |  |  |  |  |  |
| 2 | Aspartic acid | 27.56 ± 0.12 | 33.14 ± 1.53 | 33.04 ± 1.48 | 80.19 ± 0.17 | 72.24 ± 0.58 | 48.12 ± 0.96 | 40.73 ± 0.10 | 41.24 ± 2.04 | 44.28 ± 0.45 |
| 3 | Glutamic acid | 334.91 ± 3.87 | 385.16 ± 15.74 | 388.03 ± 15.10 | 197.3 ± 2.24 | 184.61 ± 1.83 | 123.73 ± 1.67 | 383.31 ± 3.25 | 522.12 ± 42.61 | 407.3 ± 2.40 |
|  | **TOTAL** | 362.47 ± 3.87 | 418.3 ± 15.82 | 421.07 ± 15.17 | 277.49 ± 2.41 | 256.85 ± 1.92 | 171.85 ± 1.92 | 424.04 ± 3.25 | 563.36 ± 42.65 | 451.58 ± 2.45 |
|  | **Polar/Neutral AA** |  |  |  |  |  |  |  |  |  |
| 4 | Threonine | 44.45 ± 0.54 | 54.35 ± 1.41 | 47.24 ± 1.05 | 31.51 ± 0.03 | 28.66 ± 0.50 | 13.98 ± 0.32 | 50.00 ± 0.34 | 68.87 ± 3.09 | 54.35 ± 2.38 |
| 5 | Serine | 28.77 ± 0.89 | 36.50 ± 1.34 | 33.84 ± 0.87 | 10.21 ± 0.06 | 10.47 ± 0.77 | 6.55 ± 0.34 | 33.56 ± 0.42 | 47.34 ± 2.04 | 34.59 ± 1.58 |
| 6 | Asparagine | 63.10 ± 0.11 | 80.38 ± 0.89 | 65.77 ± 1.24 | 6.80 ± 0.24 | 6.04 ± 0.22 | 3.10 ± 0.16 | 76.62 ± 0.48 | 107.26 ± 3.10 | 77.04 ± 2.34 |
| 7 | Glycin | 24.99 ± 0.46 | 31.40 ± 0.32 | 27.32 ± 0.98 | 17.70 ± 0.11 | 17.89 ± 0.06 | 10.48 ± 0.13 | 32.29 ± 0.03 | 45.68 ± 2.10 | 34.68 ± 0.44 |
|  | **TOTAL** | 161.31 ± 1.15 | 202.63 ± 2.16 | 174.17 ± 2.08 | 66.22 ± 0.27 | 63.06 ± 0.95 | 34.11 ± 0.51 | 192.47 ± 0.72 | 269.15 ± 5.27 | 200.66 ± 3.72 |
|  | **Non-Polar/Hydrophobic AA** |  |  |  |  |  |  |  |  |  |
| 8 | Alanine | 27.57 ± 0.16 | 32.57 ± 0.14 | 29.06 ± 1.35 | 20.58 ± 0.01 | 20.09 ± 0.00 | 12.20 ± 0.42 | 32.79 ± 0.26 | 52.30 ± 2.46 | 36.32 ± 0.77 |
| 9 | Valine | 82.22 ± 1.48 | 103.36 ± 4.32 | 98.94 ± 5.55 | 65.13 ± 1.21 | 63.22 ± 0.14 | 27.66 ± 1.23 | 125.52 ± 0.59 | 182.23 ± 9.78 | 134.14 ± 3.27 |
| 10 | Methionine | 38.14 ± 0.27 | 46.36 ± 0.53 | 43.58 ± 1.96 | 30.05 ± 0.65 | 28.06 ± 0.42 | 17.88 ± 0.63 | 51.36 ± 0.48 | 67.26 ± 3.21 | 51.59 ± 1.36 |
| 11 | Isoleucine | 43.45 ± 0.43 | 53.98 ± 3.40 | 54.46 ± 3.24 | 37.52 ± 0.14 | 35.24 ± 0.16 | 19.29 ± 0.70 | 76.27 ± 0.76 | 86.74 ± 7.44 | 78.92 ± 2.15 |
| 12 | Leucine | 146.15 ± 1.34 | 174.75 ± 4.93 | 163.35 ± 5.29 | 137.20 ± 0.08 | 131.48 ± 2.23 | 79.67 ± 1.93 | 195.48 ± 0.31 | 269.05 ± 14.26 | 194.95 ± 4.42 |
| 13 | Phenylalanine | 63.24 ± 0.08 | 77.18 ± 3.44 | 78.98 ± 4.02 | 65.38 ± 0.96 | 64.98 ± 0.67 | 35.33 ± 1.03 | 105.61 ± 0.98 | 132.06 ± 9.09 | 100.90 ± 0.21 |
| 14 | Proline | 109.70 ± 5.24 | 145.78 ± 3.84 | 138.03 ± 4.82 | 84.52 ± 2.01 | 79.55 ± 1.51 | 49.20 ± 0.62 | 151.93 ± 0.91 | 216.61 ± 10.65 | 157.08 ± 0.11 |
|  | **TOTAL** | 510.47 ± 5.63 | 633.98 ± 9.03 | 606.40 ± 10.70 | 440.38 ± 2.62 | 422.62 ± 2.82 | 241.23 ± 2.78 | 738.96 ± 1.76 | 1006.25 ± 23.81 | 753.9 ± 6.11 |
|  | **Basic AA** |  |  |  |  |  |  |  |  |  |
| 15 | Lysine | 195.08 ± 1.23 | 226.66 ± 7.32 | 216.1 ± 7.08 | 149.73 ± 1.38 | 146.12 ± 0.54 | 97.08 ± 2.20 | 252.35 ± 0.73 | 303.77 ± 19.92 | 253.86 ± 3.26 |
| 16 | Histidine | 38.04 ± 0.83 | 38.54 ± 2.27 | 45.72 ± 2.89 | 28.41 ± 0.25 | 31.99 ± 0.64 | 18.42 ± 1.38 | 55.76 ± 0.36 | 60.81 ± 6.42 | 53.35 ± 0.93 |
|  | **TOTAL** | 233.12 ± 1.49 | 265.20 ± 7.66 | 261.82 ± 7.65 | 178.14 ± 1.40 | 178.11 ± 0.84 | 115.50 ± 2.59 | 308.11 ± 0.82 | 364.58 ± 20.93 | 307.21 ± 3.39 |
|  | **Non-Proteinogenic AA** |  |  |  |  |  |  |  |  |  |
| 17 | Gamma-aminobutyric acid | 0.00 ± 0.00 | 0.00 ± 0.00 | 0.00 ± 0.00 | 0.00 ± 0.00 | 0.00 ± 0.00 | 0.00 ± 0.00 | 0.00 ± 0.00 | 0.00 ± 0.00 | 0.00 ± 0.00 |
| 18 | Ornithine | 23.85 ± 0.40 | 27.93 ± 1.09 | 27.43 ± 1.27 | 44.63 ± 0.11 | 40.94 ± 1.16 | 21.97 ± 0.64 | 40.92 ± 0.47 | 71.92 ± 3.84 | 45.99 ± 1.70 |
|  | **TOTAL** | 23.85 ± 0.40 | 27.93 ± 1.09 | 27.43 ± 1.27 | 44.63 ± 0.11 | 40.94 ± 1.16 | 21.97 ± 0.64 | 40.92 ± 0.47 | 71.92 ± 3.84 | 45.99 ± 1.70 |

**Table S4 continued.**

| **Tiroler Bergkäse PDO** | | | | | | | | | | |
| --- | --- | --- | --- | --- | --- | --- | --- | --- | --- | --- |
| **N°** | **FAA** | A_BKGU | B_BKGU | C_BKGU | D_BKGU | E_BKGU | F_BKGU | G_BKGU | H_BKGU | I_BKGU |
|  | **Phosphorylated AA** |  |  |  |  |  |  |  |  |  |
| 1 | Phospho-serine | 55.3 ± 0.34 | 21.35 ± 1.62 | 25.97 ± 0.48 | 65.91 ± 0.49 | 66.21 ± 0.57 | 60.29 ± 1.84 | 23.11 ± 2.50 | 24.17 ± 0.80 | 33.41 ± 1.58 |
|  | **TOTAL** |  |  |  |  |  |  |  |  |  |
|  | **Acidic AA** |  |  |  |  |  |  |  |  |  |
| 2 | Aspartic acid | 71.95 ± 0.45 | 34.15 ± 0.66 | 41.12 ± 0.61 | 86.04 ± 0.78 | 89.28 ± 0.27 | 82.17 ± 2.57 | 41.29 ± 3.94 | 34.22 ± 0.09 | 44.46 ± 1.44 |
| 3 | Glutamic acid | 598.65 ± 2.22 | 294.2 ± 7.31 | 342.68 ± 5.72 | 639.88 ± 0.16 | 622.18 ± 1.50 | 558.16 ± 13.85 | 388.86 ± 35.47 | 345.81 ± 3.62 | 425.05 ± 6.00 |
|  | **TOTAL** | 670.60 ± 2.26 | 328.35 ± 7.34 | 383.80 ± 5.76 | 725.92 ± 0.80 | 711.46 ± 1.53 | 640.33 ± 14.09 | 430.15 ± 35.69 | 380.03 ± 3.63 | 469.51 ± 6.17 |
|  | **Polar/Neutral AA** |  |  |  |  |  |  |  |  |  |
| 4 | Threonine | 99.01 ± 0.00 | 60.53 ± 0.60 | 67.02 ± 0.59 | 124.63 ± 0.14 | 123.84 ± 0.43 | 130.38 ± 1.17 | 46.47 ± 2.70 | 48.97 ± 0.79 | 51.83 ± 0.31 |
| 5 | Serine | 78.52 ± 0.73 | 46.78 ± 0.20 | 51.98 ± 0.96 | 100.32 ± 0.15 | 106.14 ± 0.34 | 102.38 ± 2.21 | 31.52 ± 2.34 | 35.47 ± 0.56 | 39.28 ± 0.62 |
| 6 | Asparagine | 136.08 ± 0.83 | 100.64 ± 1.09 | 103.55 ± 2.08 | 165.60 ± 2.21 | 172.22 ± 3.23 | 184.06 ± 1.72 | 89.08 ± 0.42 | 72.62 ± 1.23 | 73.40 ± 0.33 |
| 7 | Glycin | 55.04 ± 0.38 | 35.78 ± 0.19 | 38.90 ± 0.75 | 62.16 ± 0.01 | 62.05 ± 0.11 | 65.33 ± 1.63 | 31.11 ± 0.50 | 27.30 ± 0.44 | 29.75 ± 0.12 |
|  | **TOTAL** | 368.65 ± 1.16 | 243.73 ± 1.28 | 261.45 ± 2.48 | 452.71 ± 2.22 | 464.25 ± 3.28 | 482.15 ± 3.45 | 198.18 ± 3.63 | 184.36 ± 1.62 | 194.26 ± 0.78 |
|  | **Non-Polar/Hydrophobic AA** |  |  |  |  |  |  |  |  |  |
| 8 | Alanine | 59.68 ± 0.33 | 44.69 ± 0.33 | 48.32 ± 0.84 | 72.30 ± 0.15 | 69.45 ± 0.06 | 72.52 ± 1.49 | 35.39 ± 0.02 | 32.45 ± 0.65 | 33.59 ± 0.03 |
| 9 | Valine | 219.74 ± 0.02 | 123.64 ± 0.97 | 137.80 ± 1.96 | 263.88 ± 2.16 | 265.09 ± 0.68 | 263.22 ± 5.40 | 112.36 ± 9.17 | 103.54 ± 0.42 | 117.59 ± 1.47 |
| 10 | Methionine | 77.45 ± 0.38 | 47.34 ± 0.83 | 51.56 ± 0.64 | 93.26 ± 0.16 | 91.76 ± 0.39 | 89.87 ± 1.74 | 41.87 ± 1.80 | 38.78 ± 0.08 | 43.51 ± 0.02 |
| 11 | Isoleucine | 145.44 ± 0.60 | 60.90 ± 1.06 | 75.06 ± 1.47 | 178.40 ± 0.56 | 202.62 ± 0.93 | 189.87 ± 6.44 | 57.57 ± 8.10 | 50.63 ± 0.33 | 64.04 ± 1.15 |
| 12 | Leucine | 298.07 ± 1.04 | 203.80 ± 0.05 | 215.68 ± 3.06 | 334.73 ± 0.43 | 350.58 ± 0.79 | 350.19 ± 5.03 | 207.26 ± 5.94 | 168.01 ± 0.55 | 181.85 ± 0.11 |
| 13 | Phenylalanine | 161.84 ± 1.12 | 92.37 ± 0.41 | 104.53 ± 1.87 | 186.76 ± 0.72 | 190.20 ± 0.07 | 184.55 ± 4.26 | 100.65 ± 7.86 | 78.60 ± 0.30 | 92.86 ± 1.32 |
| 14 | Proline | 248.54 ± 0.72 | 151.53 ± 0.33 | 164.56 ± 1.93 | 292.01 ± 0.52 | 289.91 ± 0.95 | 298.63 ± 2.62 | 140.99 ± 7.28 | 136.19 ± 1.98 | 154.38 ± 2.28 |
|  | **TOTAL** | 1210.76 ± 1.86 | 724.27 ± 1.77 | 797.51 ± 4.87 | 1421.34 ± 2.44 | 1459.61 ± 1.74 | 1448.85 ± 11.23 | 696.09 ± 17.41 | 608.20 ± 2.24 | 687.82 ± 3.23 |
|  | **Basic AA** |  |  |  |  |  |  |  |  |  |
| 15 | Lysine | 398.59 ± 1.17 | 232.96 ± 3.34 | 257.67 ± 3.60 | 458.18 ± 0.11 | 469.18 ± 0.66 | 451.79 ± 10.68 | 216.01 ± 15.68 | 209.10 ± 2.39 | 230.75 ± 1.45 |
| 16 | Histidine | 89.27 ± 0.32 | 39.51 ± 0.06 | 46.64 ± 0.56 | 93.50 ± 0.80 | 104.43 ± 0.39 | 93.10 ± 4.03 | 34.97 ± 6.03 | 37.71 ± 0.48 | 49.09 ± 1.59 |
|  | **TOTAL** | 487.86 ± 1.21 | 272.47 ± 3.35 | 304.31 ± 3.64 | 551.68 ± 0.81 | 573.61 ± 0.77 | 544.89 ± 11.41 | 250.98 ± 16.80 | 246.81 ± 2.44 | 279.84 ± 2.15 |
|  | **Non-Proteinogenic AA** |  |  |  |  |  |  |  |  |  |
| 17 | Gamma-aminobutyric acid | 0.00 ± 0.00 | 0.00 ± 0.00 | 0.00 ± 0.00 | 6.03 ± 3.48 | 38.34 ± 0.68 | 60.32 ± 0.84 | 0.00 ± 0.00 | 0.00 ± 0.00 | 0.00 ± 0.00 |
| 18 | Ornithine | 30.39 ± 0.34 | 37.27 ± 0.26 | 35.15 ± 0.46 | 65.47 ± 0.20 | 54.26 ± 0.64 | 64.39 ± 1.36 | 47.24 ± 1.77 | 26.93 ± 0.41 | 23.36 ± 0.69 |
|  | **TOTAL** | 30.39 ± 0.34 | 37.27 ± 0.26 | 35.15 ± 0.46 | 71.5 ± 3.49 | 92.6 ± 0.93 | 124.71 ± 1.60 | 47.24 ± 1.77 | 26.93 ± 0.41 | 23.36 ± 0.69 |

**Table S4 continued.**

| **Stilfser type w/o PDO** | | | | | | | | | | |
| --- | --- | --- | --- | --- | --- | --- | --- | --- | --- | --- |
| **N°** | **FAA** | A_BATO | B_BATO | C_BATO | D_BATO | E_BATO | F_BATO | G_BATO | H_BATO | I_BATO |
|  | **Phosphorylated AA** |  |  |  |  |  |  |  |  |  |
| 1 | Phospho-serine | 25.21 ± 0.98 | 25.64 ± 0.12 | 22.02 ± 0.46 | 14.11 ± 0.50 | 13.82 ± 0.20 | 9.86 ± 0.45 | 9.47 ± 0.54 | 10.96 ± 0.60 | 16.59 ± 1.02 |
|  | **TOTAL** |  |  |  |  |  |  |  |  |  |
|  | **Acidic AA** |  |  |  |  |  |  |  |  |  |
| 2 | Aspartic acid | 15.54 ± 0.01 | 22.32 ± 0.05 | 20.07 ± 0.08 | 9.78 ± 0.16 | 9.12 ± 0.02 | 8.36 ± 0.09 | 9.69 ± 0.03 | 10.40 ± 0.29 | 13.93 ± 0.56 |
| 3 | Glutamic acid | 127.65 ± 0.93 | 233.41 ± 6.01 | 186.44 ± 5.12 | 31.72 ± 0.75 | 39.63 ± 3.11 | 35.82 ± 0.45 | 19.34 ± 0.09 | 34.11 ± 4.40 | 76.72 ± 14.93 |
|  | **TOTAL** | 143.19 ± 0.93 | 255.73 ± 6.01 | 206.51 ± 5.12 | 41.50 ± 0.59 | 48.75 ± 3.09 | 44.18 ± 0.46 | 29.03 ± 0.10 | 44.51 ± 4.41 | 90.65 ± 14.94 |
|  | **Polar/Neutral AA** |  |  |  |  |  |  |  |  |  |
| 4 | Threonine | 24.81 ± 0.27 | 33.14 ± 0.40 | 31.92 ± 0.13 | 9.47 ± 0.94 | 8.10 ± 0.13 | 8.51 ± 0.75 | 6.24 ± 0.04 | 9.15 ± 0.22 | 13.70 ± 0.62 |
| 5 | Serine | 23.66 ± 1.01 | 33.71 ± 0.30 | 30.00 ± 0.63 | 7.07 ± 0.18 | 8.19 ± 0.05 | 6.79 ± 0.6 | 8.31 ± 0.88 | 9.75 ± 0.27 | 15.19 ± 1.00 |
| 6 | Asparagine | 62.04 ± 0.07 | 65.90 ± 1.77 | 84.77 ± 1.08 | 17.24 ± 1.17 | 21.37 ± 0.45 | 16.94 ± 0.52 | 22.49 ± 0.08 | 32.81 ± 0.68 | 35.83 ± 4.85 |
| 7 | Glycin | 16.66 ± 0.02 | 34.68 ± 0.18 | 28.31 ± 0.90 | 7.32 ± 0.55 | 7.87 ± 0.13 | 6.92 ± 0.23 | 10.21 ± 0.43 | 10.42 ± 0.32 | 13.45 ± 0.66 |
|  | **TOTAL** | 127.17 ± 1.05 | 167.43 ± 1.85 | 175 ± 1.55 | 41.10 ± 1.61 | 45.53 ± 0.49 | 39.16 ± 1.12 | 47.25 ± 0.99 | 62.13 ± 0.83 | 78.17 ± 5.04 |
|  | **Non-Polar/Hydrophobic AA** |  |  |  |  |  |  |  |  |  |
| 8 | Alanine | 30.11 ± 0.19 | 56.46 ± 0.15 | 42.99 ± 1.17 | 19.13 ± 1.07 | 20.22 ± 0.23 | 15.16 ± 0.97 | 14.4 ± 1.03 | 15.87 ± 0.03 | 20.95 ± 0.74 |
| 9 | Valine | 58.46 ± 0.20 | 108.02 ± 0.87 | 82.76 ± 1.84 | 22.61 ± 1.66 | 23.57 ± 1.54 | 16.45 ± 0.88 | 25.32 ± 0.14 | 28.41 ± 0.16 | 44.33 ± 1.59 |
| 10 | Methionine | 33.80 ± 0.04 | 49.40 ± 0.65 | 36.79 ± 1.41 | 13.18 ± 0.07 | 14.50 ± 1.00 | 8.40 ± 0.81 | 11.06 ± 0.40 | 11.62 ± 0.82 | 18.47 ± 0.29 |
| 11 | Isoleucine | 20.22 ± 0.13 | 39.49 ± 0.18 | 26.97 ± 0.11 | 7.92 ± 0.39 | 8.78 ± 0.87 | 6.11 ± 0.09 | 7.90 ± 0.16 | 7.00 ± 0.30 | 11.32 ± 0.81 |
| 12 | Leucine | 134.20 ± 0.21 | 194.39 ± 0.46 | 178.81 ± 0.87 | 64.98 ± 2.93 | 63.38 ± 0.83 | 54.84 ± 1.81 | 82.40 ± 0.55 | 85.60 ± 1.70 | 110.89 ± 2.19 |
| 13 | Phenylalanine | 117.45 ± 0.41 | 160.41 ± 0.75 | 142.04 ± 0.86 | 57.15 ± 1.14 | 55.69 ± 0.52 | 41.01 ± 0.79 | 72.09 ± 0.01 | 72.33 ± 0.98 | 89.68 ± 2.24 |
| 14 | Proline | 69.99 ± 1.43 | 130.33 ± 5.49 | 93.43 ± 0.79 | 34.77 ± 0.30 | 13.45 ± 7.77 | 31.09 ± 1.72 | 45.35 ± 0.89 | 44.37 ± 0.16 | 63.68 ± 0.68 |
|  | **TOTAL** | 464.23 ± 1.54 | 738.50 ± 5.67 | 603.79 ± 2.97 | 219.74 ± 3.74 | 199.59 ± 8.09 | 173.06 ± 3.04 | 258.52 ± 1.53 | 265.20 ± 2.16 | 359.37 ± 3.75 |
|  | **Basic AA** |  |  |  |  |  |  |  |  |  |
| 15 | Lysine | 132.14 ± 0.39 | 186.00 ± 0.98 | 142.12 ± 2.28 | 71.07 ± 3.62 | 72.67 ± 2.40 | 48.19 ± 0.67 | 51.78 ± 0.91 | 46.60 ± 2.57 | 71.99 ± 2.38 |
| 16 | Histidine | 16.87 ± 0.02 | 30.53 ± 0.04 | 24.60 ± 0.42 | 9.31 ± 0.20 | 10.37 ± 0.14 | 9.66 ± 0.52 | 9.45 ± 0.30 | 8.44 ± 0.48 | 7.94 ± 0.92 |
|  | **TOTAL** | 149.01 ± 0.39 | 216.53 ± 0.98 | 166.72 ± 2.32 | 80.38 ± 3.62 | 83.04 ± 2.41 | 57.85 ± 0.85 | 61.23 ± 0.96 | 55.04 ± 2.61 | 79.93 ± 2.56 |
|  | **Non-Proteinogenic AA** |  |  |  |  |  |  |  |  |  |
| 17 | Gamma-aminobutyric acid | 0.00 ± 0.00 | 21.05 ± 1.03 | 15.07 ± 0.92 | 0.00 ± 0.00 | 0.00 ± 0.00 | 0.00 ± 0.00 | 6.33 ± 3.65 | 17.52 ± 1.65 | 0.00 ± 0.00 |
| 18 | Ornithine | 57.09 ± 0.08 | 67.80 ± 0.55 | 70.71 ± 0.08 | 34.25 ± 0.40 | 32.95 ± 0.41 | 29.30 ± 1.89 | 28.28 ± 0.29 | 32.49 ± 0.19 | 27.48 ± 0.16 |
|  | **TOTAL** | 57.09 ± 0.08 | 88.85 ± 1.17 | 85.78 ± 0.92 | 34.25 ± 0.40 | 32.95 ± 0.41 | 29.30 ± 1.89 | 34.61 ± 3.66 | 50.01 ± 1.66 | 27.48 ± 0.16 |

**Table S5: Assignment of GC-MS signal.**

| **Peak** | **Identification** | **LRI^a^** | **Method** | **Reference** |
| --- | --- | --- | --- | --- |
| 1 | 3-Methyl-Butanal | 905 | MS + LRI | (Lecanu et al., 2002) |
| 2 | Ethanol | 922 | MS + LRI | (Hayaloglu & Karabulut, 2013; Lecanu et al., 2002) |
| 3 | Biacetyl | 956 | MS + LRI | (Lecanu et al., 2002) |
| 4 | 2-Pentanone | 961 | MS + LRI | (Hayaloglu & Karabulut, 2013; Lecanu et al., 2002) |
| 5 | Dimethyl Disulfide | 1054 | MS + LRI | (Lecanu et al., 2002) |
| 6 | Hexanal | 1064 | MS + LRI | (Hayaloglu & Karabulut, 2013) |
| 7 | 2-Heptanone | 1169 | MS + LRI | (Hayaloglu & Karabulut, 2013; Lecanu et al., 2002) |
| 8 | 3-Methyl-1-Butanol | 1202 | MS + LRI | (Hayaloglu & Karabulut, 2013) |
| 9 | Acetoin | 1272 | MS + LRI | (Hayaloglu & Karabulut, 2013) |
| 10 | 2-Heptanol | 1315 | MS + LRI | (Hayaloglu & Karabulut, 2013) |
| 11 | 1-Hexanol | 1346 | MS + LRI | (Hayaloglu & Karabulut, 2013) |
| 12 | 2-Nonanone | 1375 | MS + LRI | (Hayaloglu & Karabulut, 2013) |
| 13 | Acetic Acid | 1440 | MS + LRI | (Hayaloglu & Karabulut, 2013) |
| 14 | Benzaldehyde | 1500 | MS |  |
| 15 | Propanoic Acid | 1525 | MS + LRI | (Hayaloglu & Karabulut, 2013; Lecanu et al., 2002) |
| 16 | 2,3-Butanediol | 1570 | MS + LRI | (Hayaloglu & Karabulut, 2013) |
| 17 | Isobutyric Acid | 1607 | MS + LRI | (Lecanu et al., 2002) |
| 18 | Butyric Acid | 1613 | MS + LRI | (Lecanu et al., 2002) |

**Table S5 continued.**

| 19 | Isovaleric Acid | 1654 | MS + LRI | (Hayaloglu & Karabulut, 2013) |
| --- | --- | --- | --- | --- |
| 20 | Valeric Acid | 1704 | MS + LRI | (Lecanu et al., 2002) |
| 21 | Hexanoic Acid | 1766 | MS + LRI | (Lecanu et al., 2002) |
| 22 | Octanoic Acid | 1857 | MS |  |

**Footnotes**

^a^LRI means linear retention indices (calculated from C8 to C20 n-linear alkanes with ZB-wax capillary column)

**References**

Hayaloglu, A. A., & Karabulut, I. (2013). SPME/GC-MS Characterization and Comparison of Volatiles of Eleven Varieties of Turkish Cheeses. International Journal of Food Properties, 16(7), 1630–1653. https://doi.org/10.1080/10942912.2011.587625

Lecanu, L., Ducruet, V., Jouquand, C., Gratadoux, J. J., & Feigenbaum, A. (2002). Optimization of Headspace Solid-Phase Microextraction (SPME) for the Odor Analysis of Surface-Ripened Cheese. Journal of Agricultural and Food Chemistry, 50(13).

**Table S6:** **Semi-quantification of volatile organic compounds identified by HS-SPME/GC-MS across cheese types (mean ± standard error) in ng/g.**

| **Bergkäse without PDO** | | | | | | | | | | |
| --- | --- | --- | --- | --- | --- | --- | --- | --- | --- | --- |
| **N°** | **Compounds** | A_BK | B_BK | C_BK | D_BK | E_BK | F_BK | G_BK | H_BK | I_BK |
|  | **Acid** |  |  |  |  |  |  |  |  |  |
| 1 | Acetic Acid | 365.17 ± 8.73 | 414.50 ± 19.88 | 443.47 ± 29.98 | 542.56 ± 11.93 | 626.14 ± 45.8 | 580.48 ± 83.43 | 501.88 ± 52.26 | 750.35 ± 43.50 | 698.45 ± 37.37 |
| 2 | Propanoic Acid | n.d. | n.d. | n.d. | 413.70 ± 13.54 | 179.37 ± 9.42 | 120.35 ± 12.10 | 138.08 ± 6.39 | 279.08 ± 14.54 | 875.32 ± 39.17 |
| 3 | Isobutyric Acid | 53.35 ± 1.75 | 46.79 ± 0.80 | 55.81 ± 4.38 | 26.91 ± 0.55 | 140.24 ± 6.78 | 50.67 ± 4.52 | 60.28 ± 3.51 | 104.24 ± 4.29 | 34.58 ± 2.29 |
| 4 | Butyric Acid | 577.79 ± 8.57 | 665.93 ± 24.98 | 733.39 ± 51.15 | 595.73 ± 5.44 | 917.54 ± 36.74 | 978.67 ± 81.14 | 456.12 ± 23.16 | 694.07 ± 28.41 | 544.89 ± 38.05 |
| 5 | Isovaleric Acid | 202.73 ± 6.38 | 217.62 ± 2.46 | 229.90 ± 15.28 | 187.52 ± 3.26 | 490.78 ± 25.21 | 228.31 ± 14.87 | 262.61 ± 13.23 | 383.81 ± 10.36 | 263.43 ± 12.51 |
| 6 | Valeric Acid | 5.51 ± 0.05 | n.d. | 7.87 ± 0.42 | n.d. | 9.85 ± 0.24 | n.d. | n.d. | n.d. | n.d. |
| 7 | Hexanoic Acid | 276.52 ± 2.60 | 434.99 ± 13.05 | 488.66 ± 16.59 | 334.81 ± 8.64 | 448.92 ± 17.98 | 482.66 ± 11.39 | 273.38 ± 13.77 | 384.11 ± 14.96 | 357.28 ± 16.14 |
| 8 | Octanoic Acid | 27.96 ± 0.37 | 44.09 ± 3.12 | 47.82 ± 1.54 | 56.70 ± 3.32 | 59.25 ± 2.11 | 70.81 ± 0.41 | 38.67 ± 1.43 | 46.99 ± 1.40 | 54.69 ± 1.65 |
|  | **TOTAL** | 1509.03 ± 22.40 | 1823.92 ± 59.33 | 2006.92 ± 116.15 | 2157.92 ± 45.59 | 2872.09 ± 142.98 | 2511.95 ± 203.48 | 1731.03 ± 94.22 | 2642.65 ± 100.19 | 2828.64 ± 147.18 |
|  | **Alcohol** |  |  |  |  |  |  |  |  |  |
| 9 | Ethanol | 85.48 ± 3.62 | 56.14 ± 4.20 | 32.73 ± 1.51 | 49.79 ± 1.93 | 68.13 ± 3.25 | 147.86 ± 7.50 | 57.47 ± 3.82 | 91.14 ± 3.88 | 98.63 ± 2.51 |
| 10 | 3-Methyl-1-Butanol | n.d. | n.d. | n.d. | 30.99 ± 2.01 | 22.49 ± 0.79 | n.d. | n.d. | n.d. | n.d. |
| 11 | 2-Heptanol | n.d. | n.d. | n.d. | n.d. | n.d. | n.d. | n.d. | n.d. | 41.99 ± 0.96 |
| 12 | 1-Hexanol | n.d. | n.d. | n.d. | 27.42 ± 2.44 | 178.37 ± 25.37 | 198.18 ± 30.78 | n.d. | n.d. | n.d. |
| 13 | 2,3-Butanediol | 15.28 ± 0.45 | 22.62 ± 2.39 | 22.49 ± 0.71 | 13.21 ± 0.25 | 11.04 ± 1.08 | 19.23 ± 2.85 | 12.70 ± 1.16 | 18.44 ± 0.99 | 14.24 ± 0.66 |
|  | **TOTAL** | 100.76 ± 4.06 | 78.76 ± 6.07 | 55.23 ± 2.20 | 121.41 ± 6.02 | 280.03 ± 24.71 | 365.27 ± 21.35 | 70.17 ± 3.43 | 109.58 ± 4.74 | 154.86 ± 2.21 |
|  | **Aldehyde** |  |  |  |  |  |  |  |  |  |
| 14 | 3-Methyl-Butanal | 32.76 ± 2.82 | n.d. | n.d. | 37.91 ± 2.62 | n.d. | n.d. | 60.67 ± 2.31 | 55.63 ± 1.74 | 66.76 ± 1.93 |
| 15 | Hexanal | n.d. | n.d. | n.d. | 27.09 ± 0.90 | 75.06 ± 5.55 | 100.94 ± 0.97 | n.d. | n.d. | n.d. |
| 16 | Benzaldehyde | n.d. | n.d. | n.d. | n.d. | n.d. | n.d. | n.d. | n.d. | n.d. |
|  | **TOTAL** | 32.76 ± 2.82 | n.d. | n.d. | 65.00 ± 3.44 | 75.06 ± 5.55 | 100.94 ± 0.97 | 60.67 ± 2.31 | 55.63 ± 1.74 | 66.76 ± 1.93 |
|  | **Ketone** |  |  |  |  |  |  |  |  |  |
| 17 | Biacetyl | n.d. | n.d. | n.d. | n.d. | n.d. | n.d. | n.d. | n.d. | n.d. |
| 18 | 2-Pentanone | 45.28 ± 0.19 | 50.18 ± 2.12 | 61.34 ± 3.13 | 69.48 ± 3.04 | 48.37 ± 4.89 | 175.33 ± 4.52 | 49.50 ± 1.36 | 47.40 ± 1.77 | 109.24 ± 0.76 |
| 19 | 2-Heptanone | 45.95 ± 0.30 | 89.16 ± 10.05 | 92.78 ± 10.23 | 95.55 ± 4.93 | 131.02 ± 12.70 | 634.72 ± 46.01 | 65.58 ± 3.96 | 115.92 ± 1.60 | 324.92 ± 0.12 |
| 20 | Acetoin | 131.04 ± 7.44 | 50.18 ± 1.56 | 71.00 ± 1.67 | 129.64 ± 13.43 | 103.19 ± 5.17 | 227.95 ± 7.05 | 84.95 ± 4.55 | 59.07 ± 0.30 | 171.71 ± 1.90 |
| 21 | 2-Nonanone | 0.00 ± 0.00 | 0.00 ± 0.00 | 0.00 ± 0.00 | 14.87 ± 0.61 | 16.86 ± 0.51 | 108.27 ± 12.76 | 13.86 ± 1.24 | 17.34 ± 0.26 | 58.47 ± 1.02 |
|  | **TOTAL** | 222.27 ± 7.34 | 189.51 ± 12.48 | 225.12 ± 14.97 | 309.54 ± 21.63 | 299.45 ± 18.23 | 1146.29 ± 53.51 | 213.88 ± 9.29 | 239.73 ± 2.25 | 664.35 ± 2.28 |
|  | **Sulphur compound** |  |  |  |  |  |  |  |  |  |
| 22 | Dimethyl Disulfide | n.d. | n.d. | n.d. | n.d. | n.d. | n.d. | n.d. | n.d. | n.d. |
|  | **TOTAL** | n.d. | n.d. | n.d. | n.d. | n.d. | n.d. | n.d. | n.d. | n.d. |

**Footnote**n.d. means not detected

**Table S6 continued.**

|  | **Tiroler Bergkäse PDO** | | | | | | | | | |
| --- | --- | --- | --- | --- | --- | --- | --- | --- | --- | --- |
| **N°** | **Compounds** | A_BKGU | B_BKGU | C_BKGU | D_BKGU | E_BKGU | F_BKGU | G_BKGU | H_BKGU | I_BKGU |
|  | **Acid** |  |  |  |  |  |  |  |  |  |
| 1 | Acetic Acid | 391.28 ± 12.86 | 891.85 ± 55.56 | 996.6 ± 60.48 | 504.22 ± 12.04 | 625.29 ± 116.33 | 555.77 ± 14.12 | 434.42 ± 27.77 | 552.90 ± 15.93 | 315.04 ± 57.1 |
| 2 | Propanoic Acid | n.d. | 16.89 ± 1.41 | 29.45 ± 6.66 | n.d. | n.d. | n.d. | 83.54 ± 4.45 | 19.74 ± 1.27 | 27.72 ± 2.61 |
| 3 | Isobutyric Acid | 48.21 ± 1.68 | 88.72 ± 2.46 | 119.90 ± 9.54 | 37.52 ± 2.92 | 87.70 ± 9.08 | 71.94 ± 7.27 | 30.12 ± 1.78 | 78.71 ± 3.88 | 48.93 ± 2.69 |
| 4 | Butyric Acid | 1509.61 ± 77.84 | 1075.74 ± 40.64 | 2256.40 ± 146.80 | 1042.30 ± 20.17 | 1348.51 ± 226.09 | 1155.61 ± 5.87 | 479.73 ± 32.12 | 1235.93 ± 28.63 | 1010.35 ± 11.11 |
| 5 | Isovaleric Acid | 398.66 ± 16.22 | 368.93 ± 8.79 | 834.25 ± 67.81 | 299.75 ± 14.22 | 518.60 ± 86.62 | 353.09 ± 14.00 | 246.97 ± 13.54 | 427.61 ± 16.33 | 283.48 ± 5.62 |
| 6 | Valeric Acid | 11.65 ± 0.70 | n.d. | 19.11 ± 1.31 | 7.29 ± 0.25 | 9.77 ± 1.43 | n.d. | n.d. | 11.49 ± 0.24 | 7.69 ± 0.39 |
| 7 | Hexanoic Acid | 545.47 ± 30.09 | 257.34 ± 6.32 | 1039.88 ± 79.30 | 338.61 ± 12.38 | 434.80 ± 103.09 | 264.56 ± 8.16 | 204.57 ± 14.43 | 453.06 ± 24.96 | 152.57 ± 20.22 |
| 8 | Octanoic Acid | 28.40 ± 1.46 | 31.52 ± 1.40 | 41.20 ± 3.80 | 34.95 ± 0.15 | 41.74 ± 5.70 | 37.51 ± 1.38 | 31.90 ± 2.07 | 40.05 ± 2.29 | 23.76 ± 1.21 |
|  | **TOTAL** | 2933.29 ± 135.11 | 2731.00 ± 110.76 | 5336.80 ± 336.61 | 2264.64 ± 44.49 | 3066.40 ± 547.10 | 2438.48 ± 19.98 | 1511.24 ± 93.90 | 2819.49 ± 52.65 | 1869.54 ± 73.49 |
|  | **Alcohol** |  |  |  |  |  |  |  |  |  |
| 9 | Ethanol | 48.67 ± 7.26 | 53.93 ± 1.76 | 60.02 ± 5.51 | 53.74 ± 0.71 | 59.15 ± 5.96 | 77.94 ± 0.21 | 67.90 ± 3.40 | 61.10 ± 0.72 | 68.66 ± 6.21 |
| 10 | 3-Methyl-1-Butanol | n.d. | n.d. | n.d. | n.d. | n.d. | n.d. | n.d. | n.d. | n.d. |
| 11 | 2-Heptanol | n.d. | n.d. | n.d. | n.d. | n.d. | n.d. | n.d. | n.d. | n.d. |
| 12 | 1-Hexanol | n.d. | n.d. | n.d. | n.d. | n.d. | 184.98 ± 3.53 | n.d. | 442.99 ± 26.06 | 1467.79 ± 152.65 |
| 13 | 2,3-Butanediol | n.d. | n.d. | n.d. | 9.91 ± 0.36 | n.d. | n.d. | n.d. | n.d. | n.d. |
|  | **TOTAL** | 48.67 ± 7.26 | 53.93 ± 1.76 | 60.02 ± 5.51 | 63.65 ± 0.59 | 59.15 ± 5.96 | 262.92 ± 3.32 | 67.90 ± 3.40 | 504.09 ± 25.99 | 1536.45 ± 158.84 |
|  | **Aldehyde** |  |  |  |  |  |  |  |  |  |
| 14 | 3-Methyl-Butanal | 54.37 ± 4.57 | 30.02 ± 4.86 | 46.84 ± 4.34 | 85.66 ± 2.97 | 72.83 ± 5.43 | 76.09 ± 4.47 | 23.06 ± 0.95 | n.d. | n.d. |
| 15 | Hexanal | 20.61 ± 1.14 | 25.64 ± 3.92 | 46.00 ± 2.52 | 39.22 ± 1.27 | 62.16 ± 10.06 | 54.18 ± 0.43 | 30.51 ± 2.41 | 182.11 ± 6.32 | 90.06 ± 5.12 |
| 16 | Benzaldehyde | n.d. | n.d. | n.d. | n.d. | n.d. | 87.47 ± 2.94 | n.d. | n.d. | 133.37 ± 10.14 |
|  | **TOTAL** | 74.98 ± 3.43 | 55.66 ± 8.78 | 92.84 ± 1.87 | 124.87 ± 4.23 | 134.99 ± 15.46 | 217.73 ± 1.95 | 53.57 ± 3.09 | 182.11 ± 6.32 | 223.43 ± 7.69 |
|  | **Ketone** |  |  |  |  |  |  |  |  |  |
| 17 | Biacetyl | n.d. | n.d. | n.d. | n.d. | n.d. | n.d. | n.d. | n.d. | n.d. |
| 18 | 2-Pentanone | 118.37 ± 4.94 | 92.30 ± 3.38 | 125.58 ± 12.26 | 113.20 ± 7.25 | 114.03 ± 20.91 | 87.29 ± 2.68 | 26.10 ± 1.80 | 78.50 ± 0.86 | 138.64 ± 7.02 |
| 19 | 2-Heptanone | 68.50 ± 2.82 | 74.26 ± 0.73 | 139.85 ± 6.50 | 52.67 ± 0.88 | 82.37 ± 9.02 | 78.16 ± 2.14 | 53.56 ± 2.32 | 121.11 ± 0.82 | 208.67 ± 14.91 |
| 20 | Acetoin | 192.49 ± 9.22 | 376.54 ± 7.62 | 335.93 ± 14.5 | 416.75 ± 15.47 | 496.29 ± 30.05 | 585.85 ± 10.29 | 85.65 ± 4.48 | 235.24 ± 8.46 | 244.04 ± 15.35 |
| 21 | 2-Nonanone | n.d. | n.d. | n.d. | n.d. | n.d. | n.d. | n.d. | n.d. | 41.68 ± 5.90 |
|  | **TOTAL** | 379.36 ± 12.93 | 543.10 ± 11.62 | 601.36 ± 30.27 | 582.62 ± 12.30 | 692.69 ± 13.92 | 751.29 ± 15.11 | 165.32 ± 8.54 | 434.85 ± 9.43 | 633.02 ± 1.75 |
|  | **Sulphur compound** |  |  |  |  |  |  |  |  |  |
| 22 | Dimethyl Disulfide | 41.33 ± 3.52 | n.d. | 80.70 ± 7.54 | 30.89 ± 0.74 | 35.60 ± 8.24 | n.d. | n.d. | n.d. | n.d. |
|  | **TOTAL** | 41.33 ± 3.52 | n.d. | 80.70 ± 7.54 | 30.89 ± 0.74 | 35.60 ± 8.24 | n.d. | n.d. | n.d. | n.d. |

**Footnote**n.d. means not detected

**Table S6 continued.**

| **Stilfser type without PDO** | | | | | | | | | | |
| --- | --- | --- | --- | --- | --- | --- | --- | --- | --- | --- |
| **N°** | **Compounds** | A_BATO | B_BATO | C_BATO | D_BATO | E_BATO | F_BATO | G_BATO | H_BATO | I_BATO |
|  | **Acid** |  |  |  |  |  |  |  |  |  |
| 1 | Acetic Acid | 961.11 ± 30.46 | 1145.75 ± 26.34 | 680.40 ± 20.63 | 1035.88 ± 36.96 | 1272.31 ± 96.11 | 955.22 ± 60.38 | 673.08 ± 36.02 | 915.90 ± 32.410 | 842.29 ± 20.96 |
| 2 | Propanoic Acid | 13.53 ± 0.47 | 33.97 ± 0.68 | n.d. | 19.56 ± 0.34 | 44.40 ± 4.41 | 17.61 ± 1.36 | 11.00 ± 0.33 | n.d. | n.d. |
| 3 | Isobutyric Acid | 312.24 ± 10.28 | 512.17 ± 0.93 | 80.51 ± 2.45 | 522.59 ± 10.03 | 483.61 ± 17.35 | 149.7 ± 3.07 | 413.21 ± 14.98 | 199.63 ± 2.16 | 159.81 ± 3.37 |
| 4 | Butyric Acid | 409.84 ± 10.53 | 539.06 ± 8.94 | 784.98 ± 14.70 | 343.58 ± 4.95 | 331.97 ± 11.91 | 387.40 ± 9.05 | 501.85 ± 17.87 | 495.93 ± 3.03 | 610.32 ± 6.77 |
| 5 | Isovaleric Acid | 1257.46 ± 33.22 | 2037.12 ± 5.15 | 401.68 ± 15.52 | 2489.46 ± 20.92 | 1977.90 ± 44.36 | 937.87 ± 10.50 | 1665.74 ± 35.95 | 879.42 ± 7.64 | 843.14 ± 13.91 |
| 6 | Valeric Acid | n.d. | n.d. | n.d. | n.d. | 10.95 ± 1.01 | 7.71 ± 0.53 | n.d. | n.d. | 5.35 ± 0.13 |
| 7 | Hexanoic Acid | 123.12 ± 2.01 | 150.74 ± 4.16 | 180.47 ± 2.42 | 128.55 ± 0.77 | 98.94 ± 3.98 | 106.85 ± 1.06 | 122.44 ± 0.74 | 111.55 ± 2.41 | 128.99 ± 2.44 |
| 8 | Octanoic Acid | 13.13 ± 0.24 | 18.05 ± 0.51 | 16.87 ± 0.28 | 17.72 ± 0.24 | 19.67 ± 1.29 | 18.50 ± 0.81 | 15.38 ± 0.17 | 17.79 ± 1.07 | 17.31 ± 0.17 |
|  | **TOTAL** | 3090.44 ± 78.90 | 4436.86 ± 44.73 | 2144.92 ± 52.34 | 4557.34 ± 69.00 | 4239.74 ± 164.35 | 2580.85 ± 72.40 | 3402.70 ± 104.15 | 2620.23 ± 30.62 | 2607.22 ± 40.11 |
|  | **Alcohol** |  |  |  |  |  |  |  |  |  |
| 9 | Ethanol | 378.53 ± 13.73 | 410.14 ± 6.78 | 447.16 ± 1.29 | 346.54 ± 15.05 | 364.54 ± 22.66 | 470.32 ± 11.02 | 224.02 ± 5.36 | 309.50 ± 3.87 | 330.88 ± 9.42 |
| 10 | 3-Methyl-1-Butanol | 24.84 ± 0.35 | 22.36 ± 0.58 | 24.58 ± 0.37 | 26.46 ± 0.41 | n.d. | n.d. | 12.92 ± 0.30 | 14.78 ± 10 | 15.39 ± 0.44 |
| 11 | 2-Heptanol | 12.35 ± 0.65 | 27.85 ± 1.95 | 17.30 ± 0.40 | n.d. | n.d. | 8.53 ± 0.35 | n.d. | n.d. | n.d. |
| 12 | 1-Hexanol | n.d. | 41.80 ± 2.65 | 102.70 ± 10.64 | n.d. | n.d. | n.d. | n.d. | n.d. | n.d. |
| 13 | 2,3-Butanediol | 39.45 ± 2.32 | 22.34 ± 0.86 | 59.62 ± 2.93 | 79.23 ± 2.27 | 47.99 ± 6.48 | 77.11 ± 8.95 | 35.09 ± 1.97 | 76.40 ± 5.24 | 63.80 ± 3.55 |
|  | **TOTAL** | 455.16 ± 16.71 | 524.49 ± 12.54 | 651.36 ± 9.59 | 452.22 ± 13.22 | 412.54 ± 29.13 | 555.96 ± 19.32 | 272.04 ± 7.51 | 400.67 ± 6.14 | 410.07 ± 10.73 |
|  | **Aldehyde** |  |  |  |  |  |  |  |  |  |
| 14 | 3-Methyl-Butanal | n.d. | n.d. | n.d. | n.d. | n.d. | n.d. | n.d. | n.d. | n.d. |
| 15 | Hexanal | n.d. | n.d. | n.d. | n.d. | n.d. | n.d. | n.d. | n.d. | n.d. |
| 16 | Benzaldehyde | 70.69 ± 7.45 | 81.57 ± 3.80 | 220.78 ± 6.55 | 74.04 ± 2.81 | n.d. | 21.54 ± 2.72 | 36.72 ± 2.96 | n.d. | n.d. |
|  | **TOTAL** | 70.69 ± 7.45 | 81.57 ± 3.80 | 220.78 ± 6.55 | 74.04 ± 2.81 | n.d. | 21.54 ± 2.72 | 36.72 ± 2.96 | n.d. | n.d. |
|  | **Ketone** |  |  |  |  |  |  |  |  |  |
| 17 | Biacetyl | n.d. | n.d. | n.d. | 66.07 ± 2.43 | n.d. | n.d. | 101.53 ± 2.26 | n.d. | 92.39 ± 4.34 |
| 18 | 2-Pentanone | n.d. | 9.02 ± 0.70 | 59.01 ± 1.99 | n.d. | n.d. | 10.76 ± 0.85 | n.d. | n.d. | n.d. |
| 19 | 2-Heptanone | n.d. | n.d. | n.d. | n.d. | n.d. | n.d. | n.d. | n.d. | n.d. |
| 20 | Acetoin | 47.66 ± 1.99 | 58.87 ± 1.69 | 326.30 ± 19.95 | 147.71 ± 5.39 | 42.29 ± 1.44 | 83.00 ± 2.61 | 478.97 ± 8.72 | 434.87 ± 42.79 | 604.90 ± 13.94 |
| 21 | 2-Nonanone | n.d. | n.d. | n.d. | n.d. | n.d. | n.d. | n.d. | n.d. | n.d. |
|  | **TOTAL** | 47.66 ± 1.99 | 67.89 ± 2.34 | 385.31 ± 18.36 | 213.79 ± 7.70 | 42.29 ± 1.44 | 93.75 ± 3.31 | 580.50 ± 10.96 | 434.87 ± 42.79 | 697.29 ± 16.02 |
|  | **Sulphur compound** |  |  |  |  |  |  |  |  |  |
| 22 | Dimethyl Disulfide | n.d. | n.d. | n.d. | n.d. | n.d. | n.d. | n.d. | n.d. | n.d. |
|  | **TOTAL** | n.d. | n.d. | n.d. | n.d. | n.d. | n.d. | n.d. | n.d. | n.d. |

**Footnote**n.d. means not detected
